# Comparative transcriptomics and host-specific parasite gene expression profiles inform on drivers of proliferative kidney disease

**DOI:** 10.1038/s41598-020-77881-7

**Published:** 2021-01-25

**Authors:** Marc Faber, Sophie Shaw, Sohye Yoon, Eduardo de Paiva Alves, Bei Wang, Zhitao Qi, Beth Okamura, Hanna Hartikainen, Christopher J. Secombes, Jason W. Holland

**Affiliations:** 1 Scottish Fish Immunology Research Centre, University of Aberdeen, Aberdeen, AB24 2TZ UK; 2 Centre for Genome Enabled Biology and Medicine, University of Aberdeen, Aberdeen, AB24 2TZ UK; 3 Aigenpulse.Com, 115J Olympic Avenue, Milton Park, Abingdon, OX14 4SA Oxfordshire UK; 4 Guangdong Provincial Key Laboratory of Pathogenic Biology and Epidemiology for Aquatic Economic Animal, Key Laboratory of Control for Disease of Aquatic Animals of Guangdong Higher Education Institutes, College of Fishery, Guangdong Ocean University, Zhanjiang, China; 5 Key Laboratory of Biochemistry and Biotechnology of Marine Wetland of Jiangsu Province, Yancheng Institute of Technology, Jiangsu, Yancheng, 224051 China; 6 Department of Life Sciences, The Natural History Museum, London, SW7 5BD UK; 7 School of Life Sciences, University of Nottingham, Nottingham, NG7 2RD UK; 8 Present Address: Genome Innovation Hub, The University of Queensland, Brisbane, QLD 4072 Australia

**Keywords:** Gene ontology, Protein function predictions, Sequence annotation, Disease model, Molecular evolution, Immune evasion, Infection, Infectious diseases, Lymphoid tissues, Parasitology, Pathogens, Transcription, Transcriptomics, Metabolism, Computational biology and bioinformatics, Developmental biology, Evolution, Immunology, Microbiology, Molecular biology, Physiology, Zoology, Animal physiology

## Abstract

The myxozoan parasite, *Tetracapsuloides* *bryosalmonae* has a two-host life cycle alternating between freshwater bryozoans and salmonid fish. Infected fish can develop Proliferative Kidney Disease, characterised by a gross lymphoid-driven kidney pathology in wild and farmed salmonids. To facilitate an in-depth understanding of *T.* *bryosalmonae*-host interactions, we have used a two-host parasite transcriptome sequencing approach in generating two parasite transcriptome assemblies; the first derived from parasite spore sacs isolated from infected bryozoans and the second from infected fish kidney tissues. This approach was adopted to minimize host contamination in the absence of a complete *T.* *bryosalmonae* genome. Parasite contigs common to both infected hosts (the intersect transcriptome; 7362 contigs) were typically AT-rich (60–75% AT). 5432 contigs within the intersect were annotated. 1930 unannotated contigs encoded for unknown transcripts. We have focused on transcripts encoding proteins involved in; nutrient acquisition, host–parasite interactions, development, cell-to-cell communication and proteins of unknown function, establishing their potential importance in each host by RT-qPCR. Host-specific expression profiles were evident, particularly in transcripts encoding proteases and proteins involved in lipid metabolism, cell adhesion, and development. We confirm for the first time the presence of homeobox proteins and a frizzled homologue in myxozoan parasites. The novel insights into myxozoan biology that this study reveals will help to focus research in developing future disease control strategies.

## Introduction

Proliferative Kidney Disease (PKD) is an economically and ecologically important disease that impacts salmonid aquaculture and wild fish populations in Europe and North America^1^. The geographic range of PKD is broad and recent disease outbreaks in a wide range of salmonid hosts underlines the status of PKD as an emerging disease. The occurrence and severity of PKD is temperature driven, with projections of warmer climates predicted to align with escalation of disease outbreaks in the future^2,3^.

PKD is caused by the myxozoan parasite, *Tetracapsuloides* *bryosalmonae*. *T.* *bryosalmonae* spores are released from the definitive bryozoan host, *Fredericella* *sultana*^4,5^. Following spore attachment and invasion of epidermal mucous cells, the parasite migrates through the vascular system to the kidney and other organs, including spleen and liver^6^. Extrasporogonic proliferation of *T.* *bryosalmonae* in kidney tissues elicits a chronic tissue pathology, characterised by lymphoid hyperplasia, granulomatous lesions, renal atrophy, anaemia^7,8^ and hyper secretion of immunoglobulins^9,10^. The severity and development of these hallmark symptoms of PKD are modified by a variety of biological, environmental and chemical stressors, impacting on parasite load, host immunity, and disease recovery^10,11^. Variation in induced pathology and parasite development is observed depending on the identity of salmonid hosts and *T.* *bryosalmonae* strains. This variation likely reflects host–parasite coevolutionary histories. For example, European strains of *T.* *bryosalmonae* are unable to produce viable sporogonic renal stages in introduced rainbow trout and provoke clinical PKD in these ‘novel’ hosts^5^. Despite these recent advances in understanding the host responses to PKD, the molecular basis of the host–parasite interactions that drive PKD development are currently poorly known and there are no therapeutic measures for disease control.

An increasing number of genomic, transcriptomic and targeted gene studies, mainly based on parasites from the myxosporean clade, place myxozoans in the Phylum Cnidaria^12–14^. As adaptations to parasitism, myxozoans exhibit extreme morphological simplification and drastically reduced genome sizes relative to free-living cnidarians^15,16^. Nevertheless, polar capsules homologous to the stinging nematocysts of cnidarians have been retained and are used for host attachment. Whilst myxozoans exhibit an apparent streamlining of metabolic and developmental processes compared to free-living cnidarians, not surprisingly, they have retained large numbers of proteases^18^. Likewise, low density lipoprotein receptor class A domain-containing proteins (LDLR-As) are also numerous^18,19^. This along with the apparent dominance of myxosporean lipases in infected fish suggests that host lipids may represent an important source of nutrients for myxozoans with lipid metabolic processes potentially contributing towards myxozoan virulence^18^.

*Tetracapsuloides* *bryosalmonae* belongs to the relative species-poor, early diverging myxozoan clade Malacosporea, which has retained primitive features, such as epithelial layers and, in some cases, musculature^20^. Malacosporeans alternate between fish and freshwater bryozoan hosts^17^. There are clear host-specific developmental differences, including meiosis in bryozoan hosts and morphologies of spores released from fish and bryozoan hosts^21^. Host specific differences in gene expression may provide avenues for the development of targeted future therapeutics and are an important prerequisite in understanding the parasite’s biology. However, biological characterization of myxozoans via transcriptome and genome data has for various reasons been hampered. Provision of sufficient and appropriate material may be problematic. For example, sporogonic stages (henceforth referred to as spore sacs) of *T.* *bryosalmonae* can be released from the body cavity of bryozoan hosts by dissection and can occasionally be collected in substantial quantity (e.g. hundreds of spore sacs) from infected bryozoans maintained in laboratory mesocosms^22^ or from field-collected material^3^. Indeed, purified spore sacs were previously used to develop a full-length normalized *T.* *bryosalmonae* cDNA library to generate the first Sanger sequenced batch of ESTs, available in GenBank. However, attempts to purify parasite stages from infected fish kidney tissues have been unsuccessful. Although dual RNA-Seq approaches and selective enrichment of parasite stages from host tissues can be attempted, most myxozoan genomes and transcriptomes still carry host contamination^23^. In situ expression experiments are hindered by low parasite to host tissue ratios in infected tissues and the subsequent very low coverage of parasite transcripts.

In the absence of a complete host-free *T.* *bryosalmonae* genome, we used two transcriptome assemblies, one from infected fish kidney and the second from infected bryozoan hosts, to develop an intersect transcriptome for comparative transcriptomics. After maximising parasite representation in tissue samples of both hosts, normalised cDNA libraries were created to maximise the characterisation of low abundance transcripts. The transcripts expressed in both host transcriptomes were retrieved in the intersect transcriptome and coupled with a novel RT-qPCR assay to assess host-specific expression profiles of parasite transcripts of interest. Our rationale was that different expression profiles are likely to indicate the relative importance of parasite genes and proteins in each host. We particularly focused on proteins linked to putative virulence mechanisms, including; cell/tissue invasion, immune evasion, protein/lipid metabolism, cell–cell communication, and development^15,18,24–27^. We hypothesised that *T.* *bryosalmonae* uses different virulence strategies in the two hosts and tested this by comparative analysis of the expression profiles of candidate key virulence genes implicated in nutritional, metabolic, virulence and developmental activities. Our approach provides the first transcriptome datasets for *T.* *bryosalmonae* and affords valuable insights into malacosporean nutritional and metabolic processes and potential virulence candidates. We identify multiple members of key gene families that may be involved in differential exploitation strategies in different hosts. These data have also revealed, for the first time in myxozoans, transcripts encoding homeobox proteins and a frizzled homologue, with the latter implying that a Wnt signalling pathway could be present in myxozoans.

## Results

### Transcriptome assembly and identification of intersect contigs

Prior to cDNA synthesis and library construction, total RNA from bryozoan-derived *T.* *bryosalmonae* spore sacs was confirmed to be of high quality with 28S rRNA/18S rRNA ratios being ≥ 1.4 (Supplementary Fig. S1). Typically, 28S rRNA/18S rRNA ratios of ≥ 1.0 correspond to RIN values higher than 8.0 (from Agilent Bioanalyser profiles)^28^. Sequencing of the normalized cDNA library from bryozoan-derived *T.* *bryosalmonae* spore sac RNA generated a total of 164,929,000 paired-end sequenced reads with 154,001,594 paired reads remaining after quality filtering and adapter trimming. Remaining reads were assembled into 48,153 contigs. 87.7% of filtered reads could be re-aligned to the assembly using Bowtie software.

Importantly, Tryptic Soy Agar (TSA) plates from kidney swabs of all fish used in this study did not reveal the presence of bacterial pathogens. Fish kidney total RNA prior to cDNA synthesis and library construction was determined to be of very high quality (28S rRNA/18S rRNA = 1.9; Supplementary Fig. S2). Sequencing of *T.* *bryosalmonae* infected trout kidney RNA generated 160,556,000 paired-end sequenced reads with 154,576,737 paired reads remaining after sequence trimming. Further sequential filtering was carried out by aligning remaining reads to a draft rainbow trout (*Oncorhynchus* *mykiss*) genome^29^ and a multi-sourced rainbow trout transcriptome database^30^. In total, 277,422,293 reads mapped to rainbow trout genome and transcriptome datasets. The remaining 31,737,181 reads were assembled into 81,035 contigs. 82.9% of filtered reads could be re-aligned to the assembly using Bowtie software.

BLAST analysis of the fish kidney-derived transcriptome assembly against a *T.* *bryosalmonae* genome sequence dataset (H. Hartikainen, B. Okamura, unpublished data) resulted in 5384 matches (6.6% of the fish kidney-derived transcriptome). Only 191 contigs were filtered with the *Thelohanellus* *kitauei* genome assembly with all 191 also being filtered with the *T.* *bryosalmonae* genome. Owing to the low number of contigs filtered using the *T.* *kitauei* genome assembly due to the high level of sequence divergence between malacosporean and myxosporean parasites, only the *T.* *bryosalmonae* genome sequence dataset was subsequently used to filter parasite contigs from the spore sac-derived transcriptome assembly, yielding 21,720 matches (45.1% of the spore sac-derived transcriptome). Reciprocal BLAST analysis of the bryozoan parasite-filtered transcriptome against the trout parasite-filtered transcriptome yielded 7362 matches and 14,358 mismatches, whilst the converse revealed 4723 matches and 661 mismatches. The 7362 contig matches were defined as the intersect transcriptome. Assembly statistics are shown in Table 1. BUSCO scores are based on the percentage complete genes found in each assembly using the core Eukaryota gene set. Scores in pre parasite-filtered assemblies are likely to be attributed to both parasite and host contamination. Initial annotation, based on BLASTX against the nr NCBI database was used to partition each transcriptome dataset into transcripts according to homology with; eukaryotes, bacteria, archaea, and viruses. Remaining contigs with *E* values above the 10^–3^ threshold were deemed as unknown/no hit.

**Table 1:** Assembly statistics for sequenced *transcriptome* datasets pre- and post-parasite filtering with *T.* *bryosalmonae* genome sequence data.

Metric	Bryozoan pre-parasite filtered	Bryozoan parasite-filtered	Trout pre-parasite filtered	Trout parasite-filtered	Intersect
Contig no.	48,153	21,720	81,035	5384	7362
Total length (bp)	33,438,146	19,352,639	34,355,816	4,866,784	9,762,738
N50 (bp)	1350	1772	837	1415	2479
GC content	41.89%	38.73%	41.50%	31.28%	33.08%
BUSCO Completeness	74.6%	66.7%	41.6%	41.6%	50.1%

**Figure 1:**
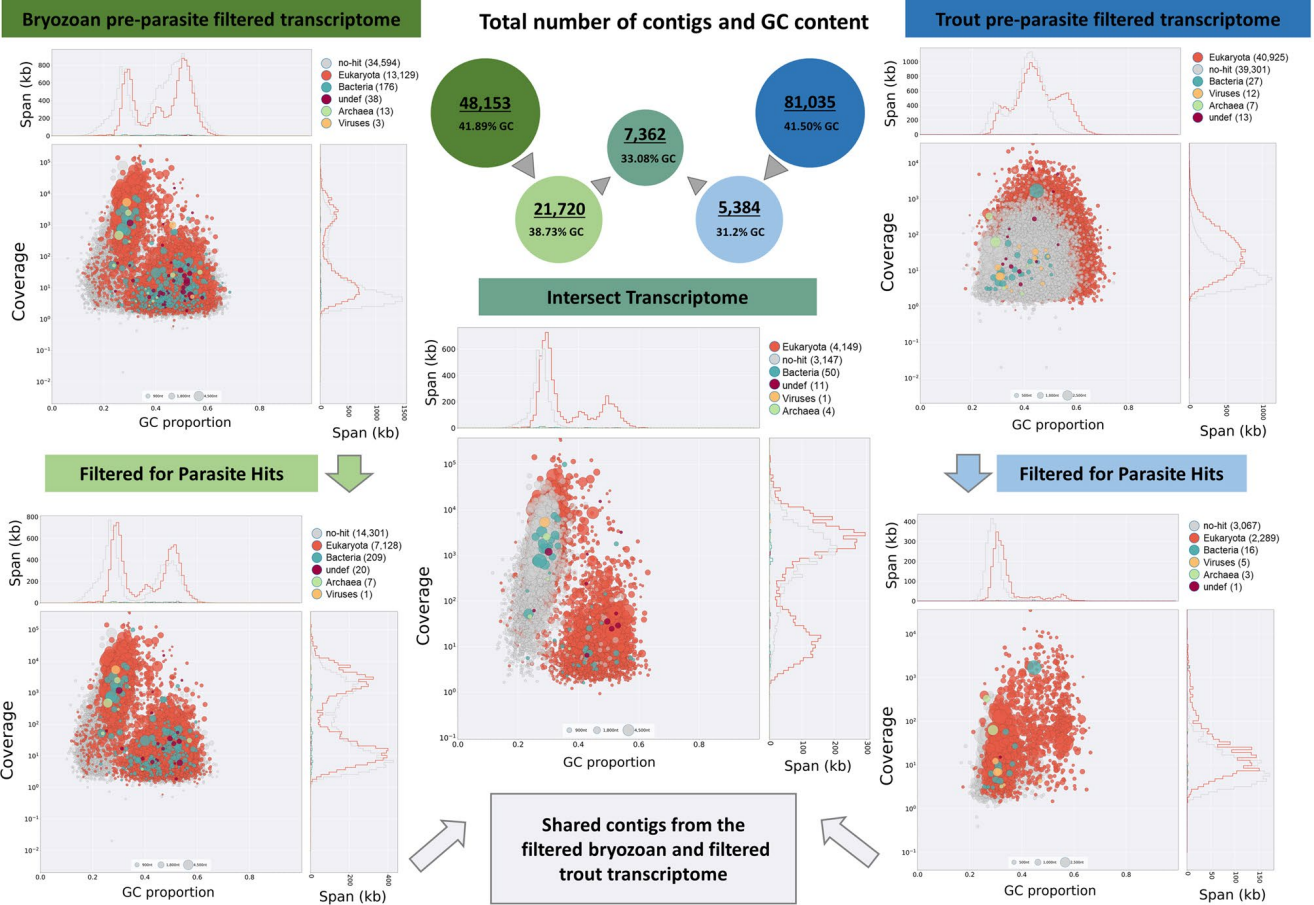
Generation of the *T.* *bryosalmonae* intersect transcriptome from spore sacs and fish kidney tissues. The contigs derived from each host assembly were filtered against the *T.* *kitauei* genome and/or a *T.* *bryosalmonae* genome sequence dataset to remove host contamination. The intersect transcriptome was generated by BLASTing the bryozoan parasite-filtered contigs against the trout parasite-filtered contigs, resulting in 7362 predicted contigs. The graphs show a BlobPlot of each contig assembly by phylum, with contigs positioned on the X-axis based on their GC proportion and on the Y-axis based on the sum of coverage. Sequence contigs in the assembly are depicted as circles, with diameter scaled proportional to sequence length and coloured by taxonomic annotation.

From the blob plot profiles, the span (Kb) and coverage of AT rich transcripts were of a similar level in bryozoan pre-filtered, bryozoan parasite-filtered and intersect transcriptomes with a marked decrease in the level of non-AT rich transcripts. AT rich transcripts were also enriched and non-AT rich transcripts markedly reduced in the trout parasite filtered transcriptome, albeit with lower span and coverage levels of the AT rich transcripts relative to the bryozoan parasite filtered and intersect transcriptomes (Fig. 1).

Pre- and parasite-filtered transcriptomes and the final intersect transcriptome are available via figshare (https://doi.org/10.6084/m9.figshare.11889672).

### Transcriptome annotation, gene ontology and MEROPS analysis

**Figure 2:**
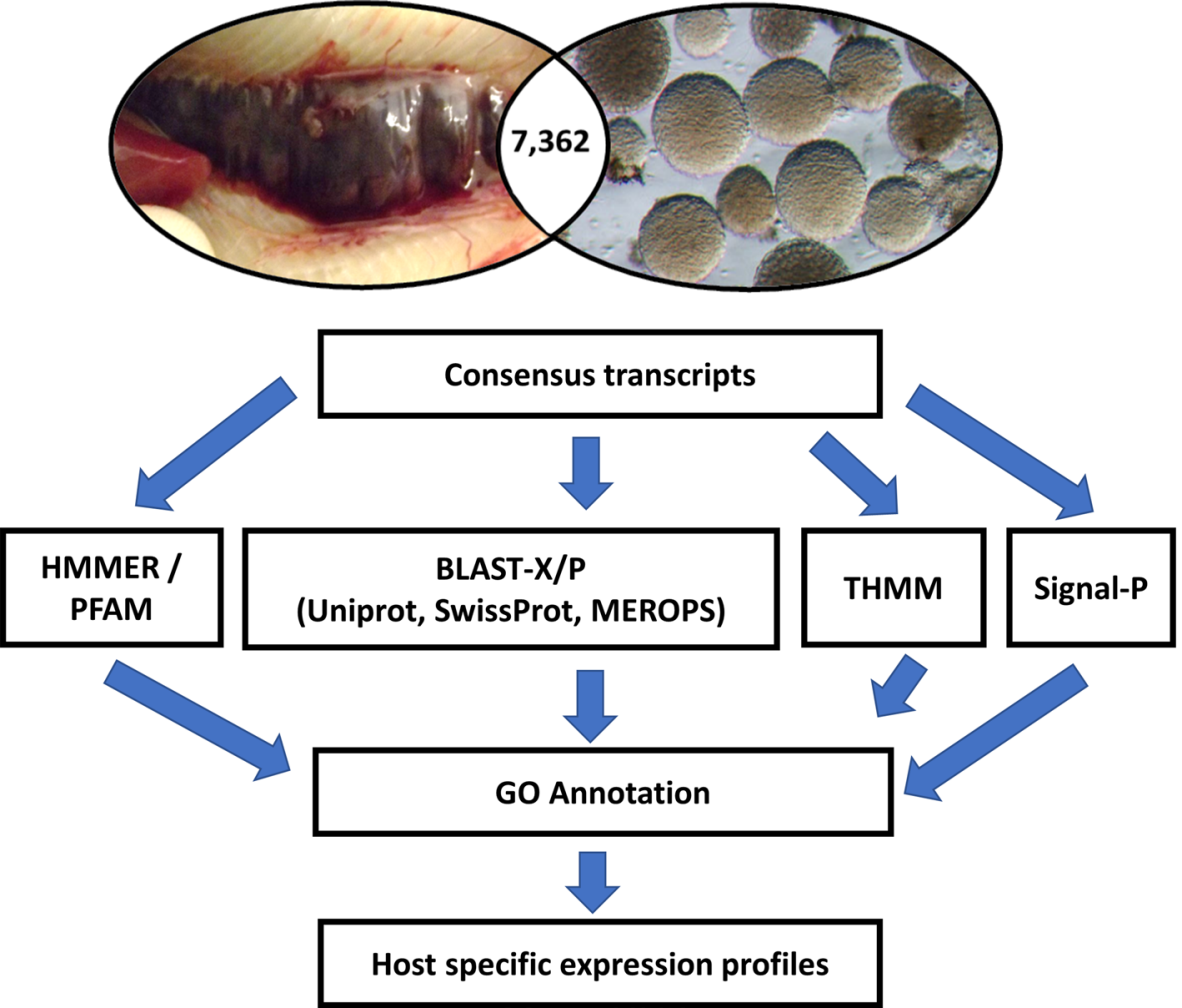
Schematic diagram of the protein annotation process based on the intersect transcriptome from spore sacs and fish kidney tissues. Protein predictions were submitted to the signalP, TMHMM, HMMER and PFam databases and BLAST analysis. Swiss-Prot matches were mapped to Gene Ontology, EggNOG and KEGG classes. Parasite contigs associated with virulence, metabolism, and development were selected and host-specific expression profiles characterised.

**Figure 3:**
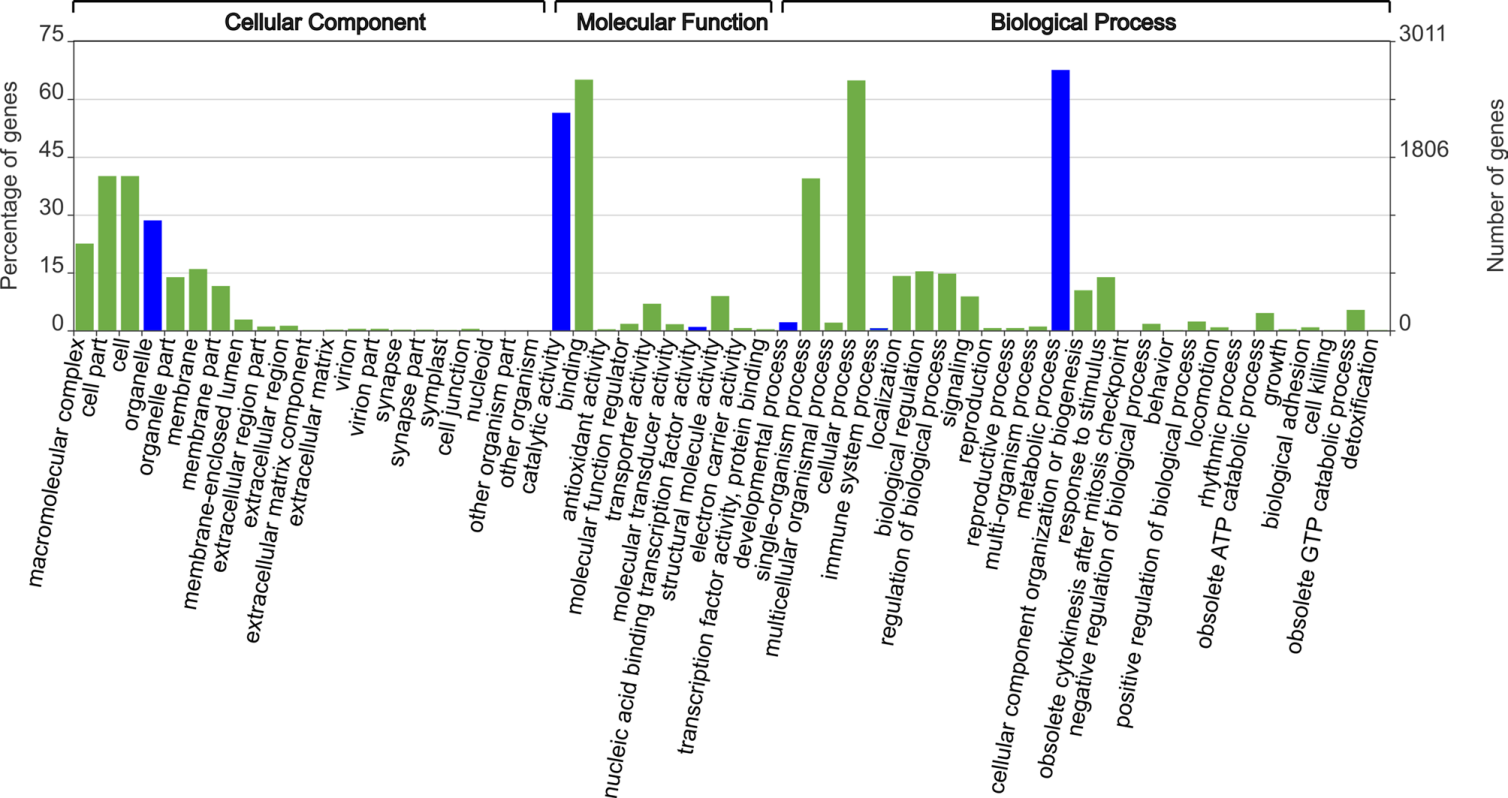
Level 2 Gene Ontology (GO) analysis of *T.* *bryosalmonae* intersect transcriptome. Functional annotation of the 4015 annotatable contigs were assigned to 9107 GO terms and grouped based on their predicted properties and function. The x-axis displays the GO terms selected from the GO trees. The left and right y-axes correspond to percentage gene number and actual gene number per selected GO term respectively. GO terms of relevance to further characterisation in this study are highlighted in blue.

Protein prediction and sequence annotation was performed on the intersect transcriptome as shown in Fig. 2. In total, 5432 contigs were homologous to protein sequences in the NCBI database (73.8%) with 1930 (26.2%) deemed as having no homology to known proteins. Of the 5432 contigs, 58% (3150 contigs) exhibited high homology (*E* value < 10^–50^) and 42% (2281 contigs) with matches between *E* values of 10^–5^ and 10^–50^ (Supplementary Table S1). 4015 were assigned to 9107 GO terms^31^. Predicted GO annotations were divided into 3 groups based on GO terms: (1) biological process (3526), (2) cellular component (1962), and (3) molecular function (3619). The GO term frequencies were plotted using the Web Gene Ontology Annotation Plot 9 (WEGO, version 2.0)^32^ for the GO-tree level 2, based on their properties and function (Fig. 3) and listed for GO-tree level 2, 3 and 4 (Supplementary Table S2). Of the annotatable and unknown protein sequences, 143 and 84 were predicted to contain a signal peptide respectively (Supplementary Tables S1, S5).

**Figure 4:**
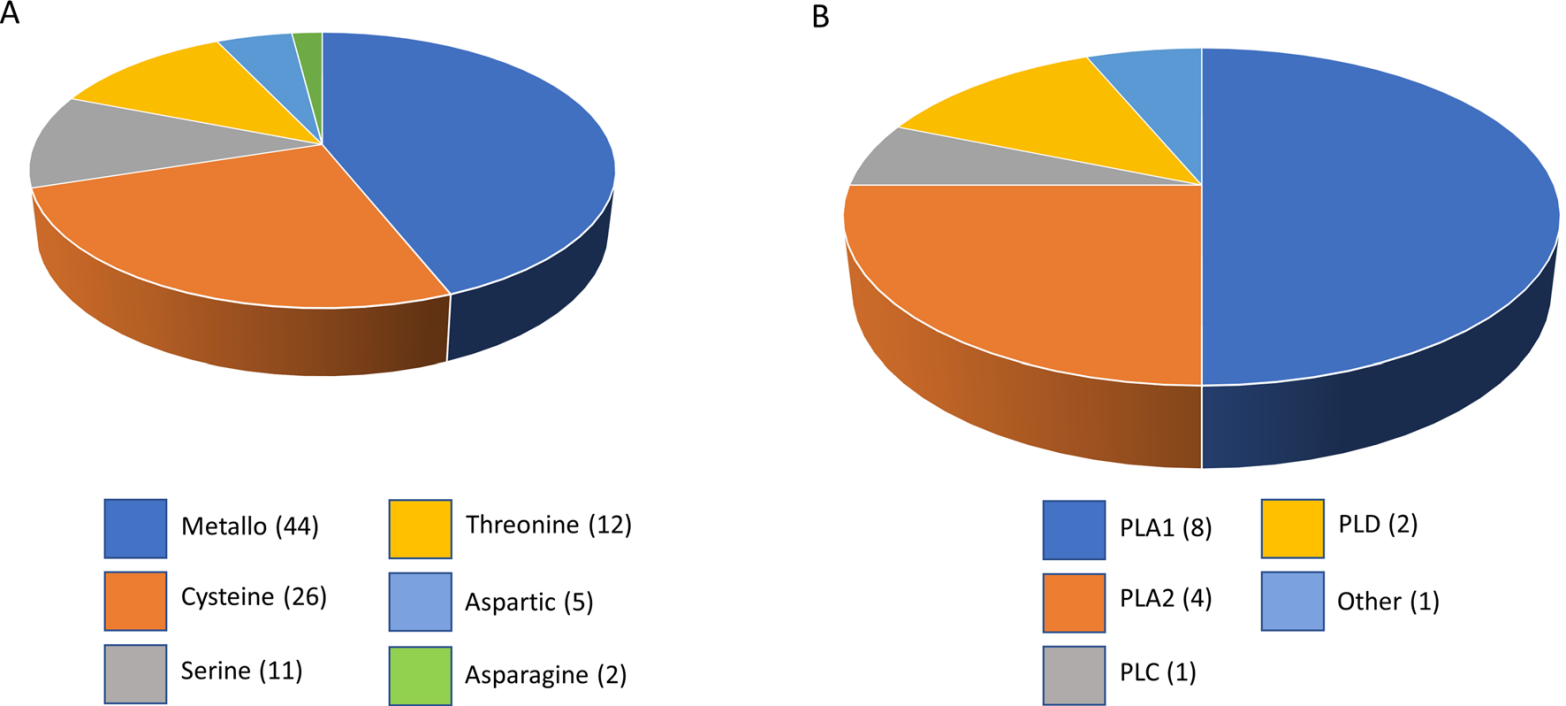
Representation in the intersect transcriptome of (**A**) protease super-families predicted by MEROPS and (**B**) manual annotated and categorised phospholipases (PL). The different colours represent different protease and lipase families.

To detect proteases in the intersect transcriptome, all contigs were analysed using the MEROPS database. A total of 100 proteases homologous to metallo- (44) and cysteine (26) proteases, threonine (12), serine (11), aspartate (5) and asparagine proteases (2) were recovered (Supplementary Table S3; Fig. 4A). All contigs predicted to be lipases or involved in lipid binding based on GO annotation were further manually annotated using BLAST and PFAM. A total of 16 lipases and 22 contigs homologous to lipid binding proteins could be identified (Supplementary Table S4; Fig. 4B). Of the lipases all, but one, were homologous to phospholipase family members. Specifically, the detected families were phospholipase A1 (8), A2 (4), C (2) and D (1) and a single contig was homologous to the hormone sensitive lipase, lipase E. Two key lipid regulatory enzymes were also uncovered, namely phosphatidate phosphatase and diacylglycerol kinase.

### Host specific gene expression

**Figure 5:**
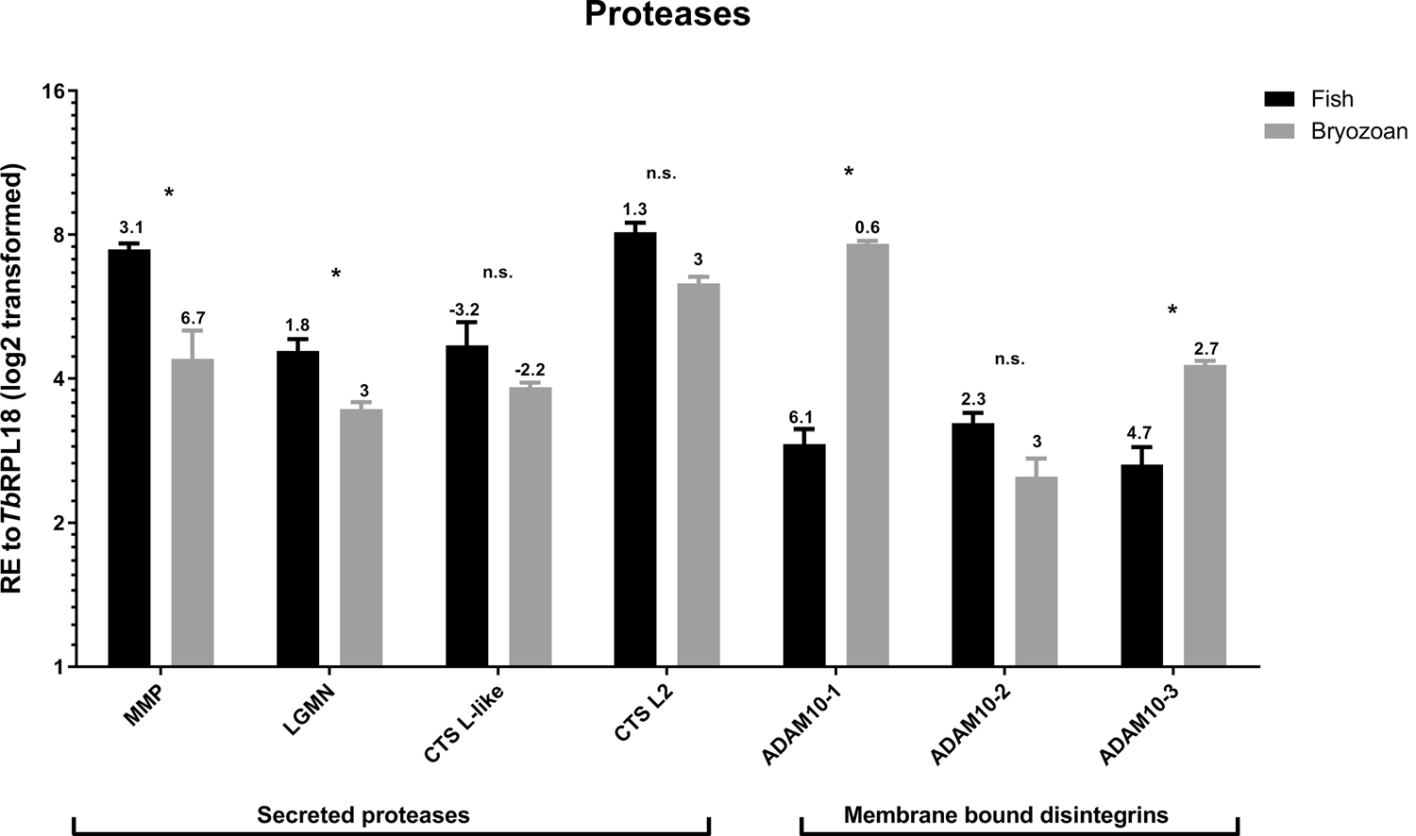
Average relative gene expression in infected bryozoan and infected fish kidney tissues of representative *T.* *bryosalmonae* proteases. All gene values were normalized to *Tb*RPL18. Δ*Cq* values, stated above each bar, represent the expression level of genes of interest relative to *Tb*RPL18 in each infected host with values > 1 indicating lower expression and values < 1 indicating higher expression relative to *Tb*RPL18. *P < 0.05. Black bars = infected fish, grey bars = infected bryozoan. Gene abbreviations: matrix metalloprotease 13 (MMP), Legumain (LGMN), Cathepsin L-like (CTLS-like), Cathepsin L2 (CTS L2) and Disintegrin and metalloproteinase domain-containing protein (ADAM)10-like-1/2/3.

**Figure 6:**
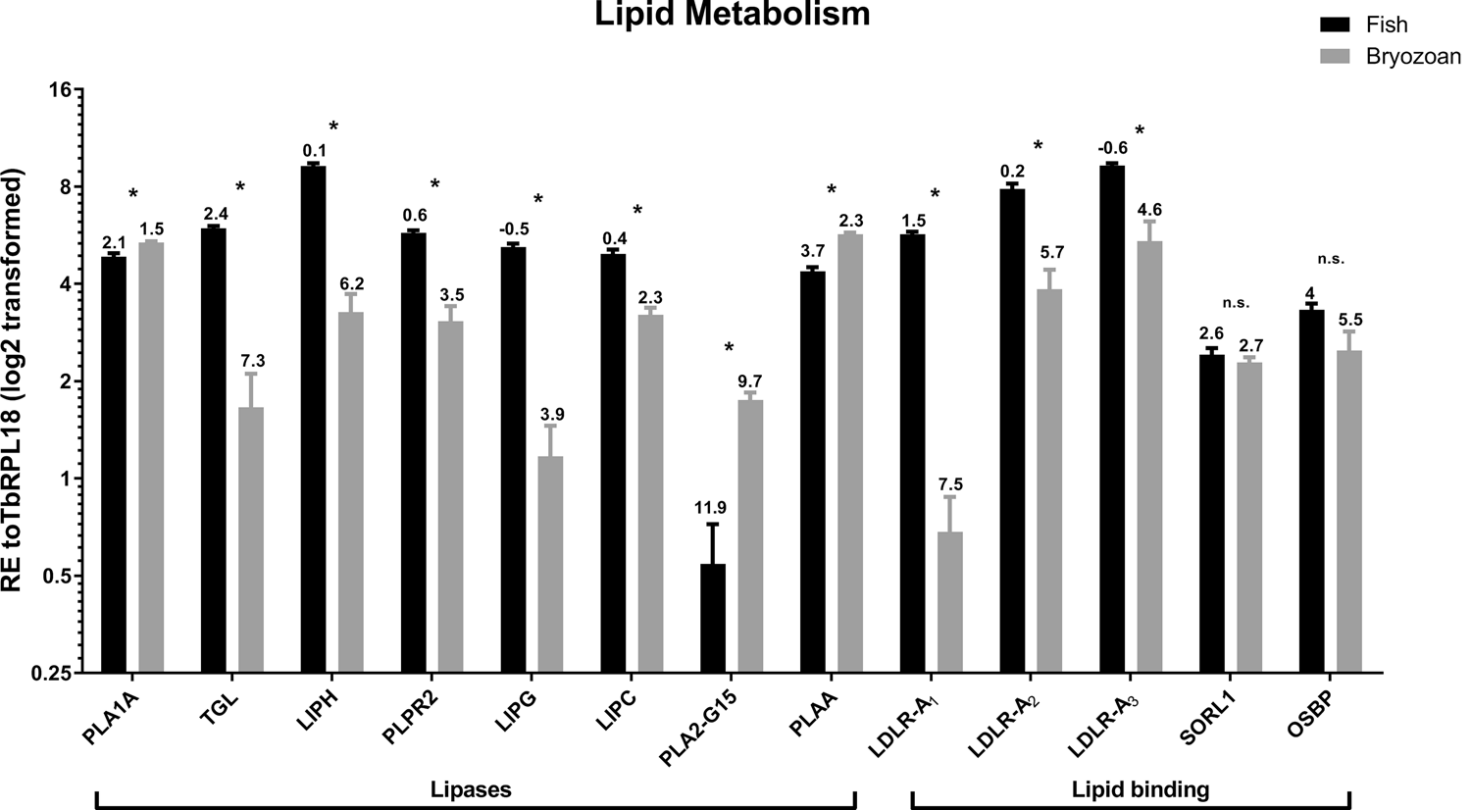
Average relative gene expression in infected bryozoan and infected fish kidney tissues of representative *T.* *bryosalmonae* genes involved in lipid metabolism. All gene values were normalized to *Tb*RPL18. Δ*Cq* values, stated above each bar, represent the expression level of genes of interest relative to *Tb*RPL18 in each infected host with values > 1 indicating lower expression and values < 1 indicating higher expression relative to *Tb*RPL18. *P < 0.05. Black bars = infected fish, grey bars = infected bryozoan. Gene abbreviations: phospholipase A1 member A-like (PLA1A), Triacyl glycerol lipase (TGL), Lipase Member H-like (LIPH), Pancreatic lipase-related protein 2 (PLPR2), Endothelial lipase (LIPG), Hepatic triacyl glycerol lipase (LIPC), Group XV phospholipase A2 (PLA2-G15), Phospholipase A-2-activating protein (PLAA), Low-density lipoprotein receptor class A-like protein 1/2/3 (LDLR-A1/2/3) , Sortilin-related receptor (SORL1) and Oxysterol-binding protein 2-like (OSBP).

**Figure 7:**
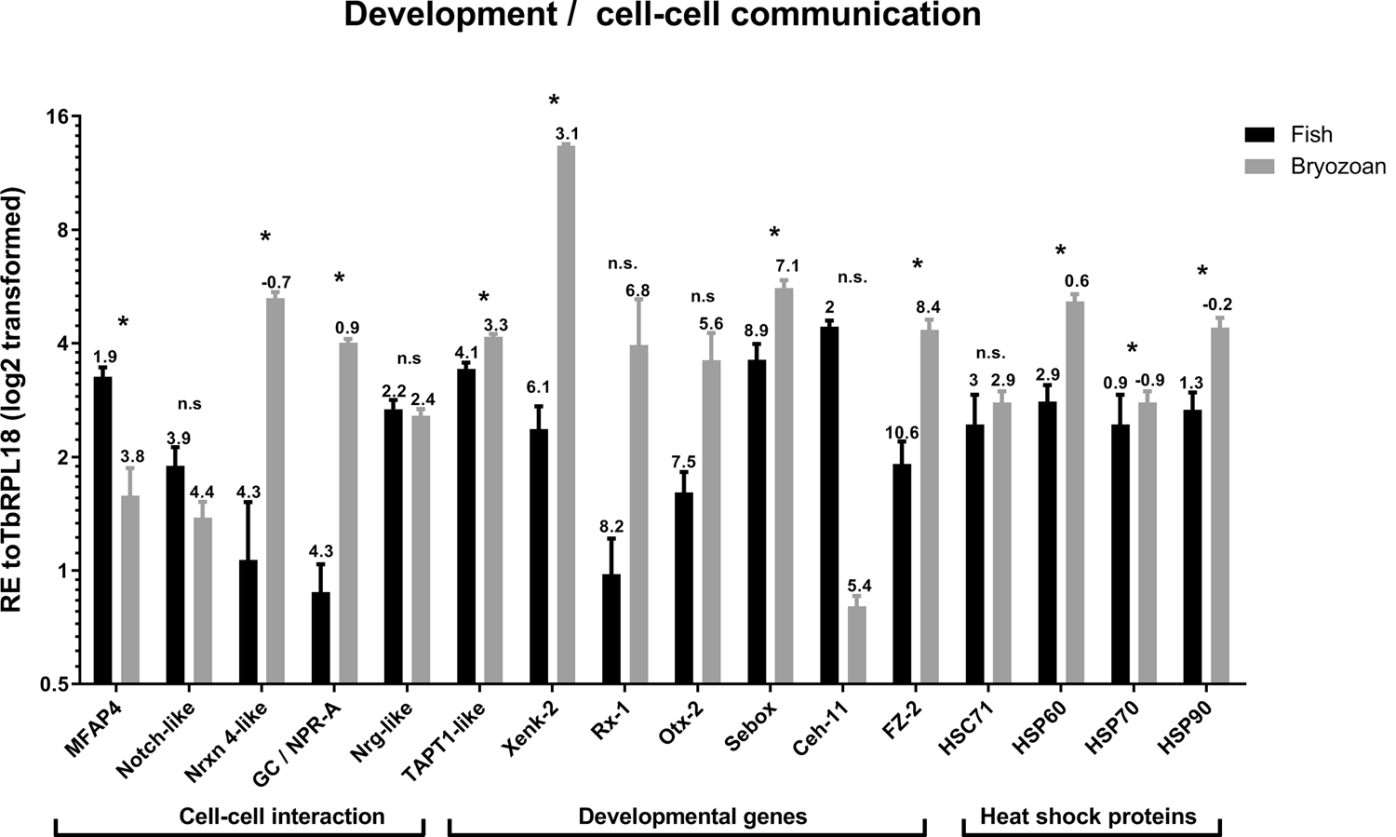
Average relative gene expression in infected bryozoan and infected fish kidney tissues of representative *T.* *bryosalmonae* genes involved in development and cell–cell communication. All gene values were normalized to *Tb*RPL18. Δ*Cq* values, stated above each bar, represent the expression level of genes of interest relative to *Tb*RPL18 in each infected host with values > 1 indicating lower expression and values < 1 indicating higher expression relative to *Tb*RPL18. *P < 0.05. Black bars = infected fish, grey bars = infected bryozoan. Gene abbreviations: microfibril-associated glycoprotein 4 (MFAP4), Notch-like protein (Notch-like), Neurexin 4-like (Nrxn 4-like), Guanylate cyclase A/atrial natriuretic peptide receptor A (GC/NPR-A), neuroglian-like protein (Nrg-like), Transmembrane anterior posterior transformation protein 1-like (TAPT1-like), Homeobox protein Xenk-2 (Xenk-2), retinal homeobox-1 (Rx-1), homeobox protein OTX2 (Otx-2), homeobox protein Sebox (Sebox), homeobox protein Ceh-11 (Ceh-11), Frizzled receptor-2 (FZ-2), heat shock protein 60 (HSP60), heat shock cognate 71 kDa protein (HSC71), heat shock protein 70 (HSP70) and heat shock protein 90 (HSP90).

**Figure 8:**
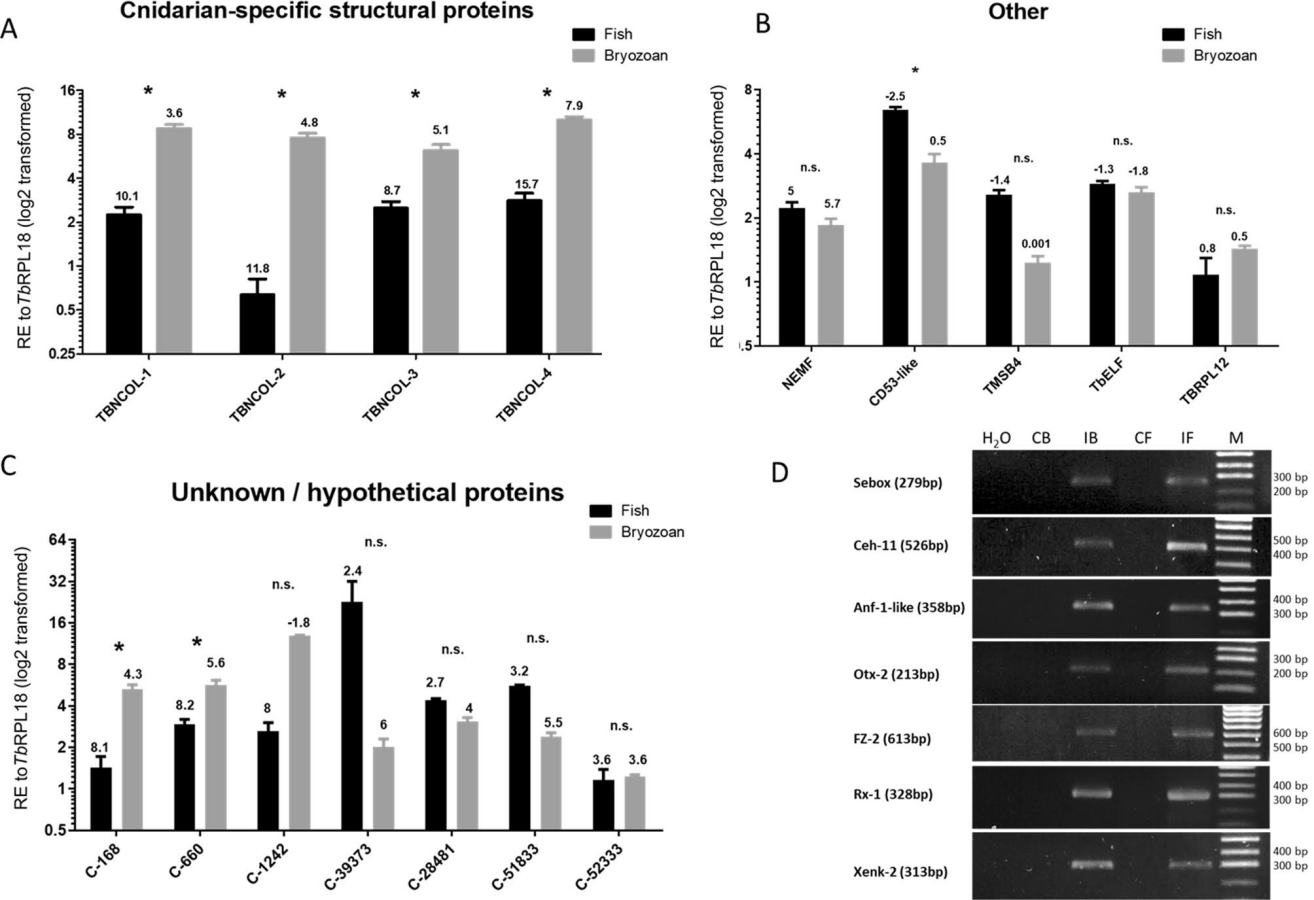
Average relative gene expression in infected bryozoan and infected fish kidney tissues of; (**A**) Cnidarian-specific structural proteins, (**B**) reference and immune-related proteins, (**C**) unknown proteins. All gene values were normalized to *Tb*RPL18. Δ*Cq* values, stated above each bar, represent the expression level of genes of interest relative to *Tb*RPL18 in each infected host with values > 1 indicating lower expression and values < 1 indicating higher expression relative to *Tb*RPL18. *P < 0.05; ***P < 0.001. Black bars = infected fish, grey bars = infected bryozoan. (**D**) PCR profile showing the presence of Sebox, Ceh-11, Anf-1-like, Otx-2, FZ-2, Rx-1 and Xenk-2 in negative control (H_2_0), uninfected bryozoan and fish kidney controls respectively (CB and CF) and *T.* *bryosalmonae*-infected bryozoan and fish genomic DNA respectively (IB and IF). M = Molecular weight marker. Full-length gel images are presented in Supplementary Fig. S3. Gene abbreviations: putative group 1 minicollagen-1/2/3/4 (TBNCOL-1/2/3/4), nuclear export mediator factor (NEMF), Thymosin beta 4-like protein (TMSB4), Tetraspanin CD53-like (CD53-like), *T.* *bryosalmonae* elongation factor alpha (*Tb*ELFa) and *T.* *bryosalmonae* ribosomal protein L12 (*Tb*RPL12), Unknown proteins C-168, C-660, C-1242, C39373, C-28481, C-51833 and C-52333.

The relative expression of 53 selected *T.* *bryosalmonae* genes was analysed to compare expression profiles in infected bryozoan and infected rainbow trout kidney tissue cDNAs (Figs. 5, 6, 7, 8). No expression of any *T.* *bryosalmonae* gene was evident in control uninfected bryozoan and fish kidney cDNA samples or in -RT controls from uninfected/infected hosts. Three previously known *T.* *bryosalmonae* reference genes were tested, and the mean quantification cycle (*Cq*) calculated for each host (fish/bryozoan) were: *Tb*RPL18 (20.00/24.51), *Tb*RPL12 (20.77/25.03) and *Tb*ELFα (18.73/23.74). Similarly, uninfected/infected fish kidney and bryozoan cDNA samples yielded a mean Cq value of 11.02 and 14.58 for rainbow trout and bryozoan ELFα respectively (Marc N. Faber unpublished data).

Coefficient of variation (CV%) values were similar for all three reference genes (*Tb*RPL12 = 9.39; *Tb*RPL18 = 10.09; *Tb*ELFα = 11.65). This implies that *Tb*RPL12 and *Tb*RPL18 were slightly more stable as reference genes than *Tb*ELFα with primer efficiency of *Tb*RPL18 amplification (1.94; 97%) being slightly higher than that for *Tb*RPL12 (1.90; 95%). Relative expression normalised to each reference gene individually revealed the same repertoire of significant and non-significant genes. Thus, gene expression data, overall, are presented normalised to *Tb*RPL18, which has been used to determine *T.* *bryosalmonae* load in trout kidney samples in previous studies^33,34^. Δ*Cq* values were calculated representing the expression level of genes of interest compared to *Tb*RPL18 in each infected host with values > 1 indicating lower expression and values < 1 indicating higher expression compared to *Tb*RPL18 (Figs. 5, 6, 7,8). Fold differences in gene expression in infected fish relative to infected bryozoans were assessed for each gene of interest and presented as fold differences in gene expression in Supplementary Table S5. Gene names were assigned according to the closest BLAST search using protein similarity search based on the manually annotated UniProtKB/Swiss-Prot and UniProtKB/Swiss-Prot isoforms databases (https://www.ebi.ac.uk/Tools/sss/fasta/). Highest BLAST hits are listed alongside predicted functional domains (Supplementary Table S5).

19 genes exhibited significantly higher expression levels in infected bryozoan samples relative to infected fish kidney samples, namely: disintegrin and metalloproteinase domain-containing protein (ADAM)10–1 (*P* = 0.029), ADAM10-3 (*P* = 0.029), Phospholipase A1 member A (PLA1A) (*P* = 0.029), Phospholipase A-2-activating protein (PLAA) (*P* = 0.029), Group XV phospholipase A2 (PLA2-G15) (*P* = 0.029), Frizzled receptor-2 (FZ-2) (*P* = 0.029), Guanylate cyclase A/atrial natriuretic peptide receptor A (GC/NPR-A) (*P* = 0.029), heat shock protein (HSP)60 (*P* = 0.029), HSP90 (*P* = 0.029), Neurexin 4 (Nrxn4)-like (*P* = 0.029), Homeobox protein Sebox (Sebox) (*P* = 0.029), Transmembrane anterior posterior transformation protein 1 (TAPT1)-like (*P* = 0.029), *T.* *bryosalmonae* minicollagen-1 (TBNCOL-1) (*P* = 0.029), TBNCOL-2 (*P* = 0.029), TBNCOL-3 (*P* = 0.029), TBNCOL-4 (*P* = 0.029), Contig (C)-660 (*P* = 0.029), C-168 (*P* = 0.029) and HSP70 (*P* = 0.029).

In contrast, 12 genes, namely; Matrix metalloprotease 13 (MMP) (*P* = 0.004), LDLR-A1 (*P* = 0.029), LDLR-A2 (*P* = 0.029), LDLR-A3 (*P* = 0.029), Legumain (LGMN) (*P* = 0.029), Hepatic triacyl glycerol lipase (LIPC) (*P* = 0.029), Endothelial lipase (LIPG) (*P* = 0.029), Lipase Member H (LIPH) (*P* = 0.029), Pancreatic lipase-related protein 2 (PLPR2) (*P* = 0.029), Triacyl glycerol lipase (TGL) (*P* = 0.029), Microfibril-associated glycoprotein 4 (MFAP4) (*P* = 0.029), and Tetraspanin CD53 (CD53)-like (*P* = 0.029), exhibited significantly higher expression levels in infected fish kidney samples relative to infected bryozoan samples.

No significantly different expression between the two hosts could be detected for 20 genes, namely; ADAM10-2 (*P* = 0.057), Cathepsin L (CTSL)-like (*P* = 0.100), CTSL2 (*P* = 0.100), Oxysterol-binding protein 2 (OSBP) (*P* = 0.114), Sortilin-related receptor (SORL1) (*P* = 0.343), Homeobox protein Ceh-11 (Ceh-11) (*P* = 0.100), HSC71 (*P* = 0.886), Notch-like (*P* = 0.114), Neuroglian-like protein (Nrg-like) (*P* = 0.486), retinal homeobox-1 (Rx-1) (*P* = 0.057), Homeobox protein OTX2 (Otx-2) (*P* = 0.057), Homeobox protein Xenk-2 (Xenk-2) (*P* = 0.057), Nuclear export mediator factor (NEMF) (*P* = 0.200), Thymosin beta 4-like protein (TMSB4) (*P* = 0.100), *T.* *bryosalmonae* elongation factor alpha (*Tb*ELFa) (*P* = 0.200), *T.* *bryosalmonae* ribosomal protein L12 (*Tb*RPL12) (*P* = 0.343), C-1242 (*P* = 0.100), C-39373 (*P* = 0.100), C-28481 (*P* = 0.100), C-51833 (*P* = 0.100) and C-52333 (*P* = 0.700).

However, there was a limited availability of biological replicates for infected bryozoan material restricted statistical analysis in some cases (where n = 3) and may underly the lack of significant differences (by Mann Whitney U test) for the genes; CTSL-like, Ceh-11, Otx-2, Rx-1, Xenk-2, TMSB4, C-39373, C-1242 , C28481, and C51833.

### Presence of frizzled receptor and homeobox genes in ***T. bryosalmonae*** genomic DNA

To further support the presence of homeobox and FZ-2 genes in *T.* *bryosalmonae*, primers were designed to specifically amplify genomic DNA from uninfected and infected hosts. PCR analysis detected specific bands, in infected but not in uninfected host tissues, of the correct predicted size and sequence verified for genes; Anf-1-like (358 bp), Sebox (279 bp), Ceh-11 (526 bp), Otx-2 (213 bp), FZ-2 (613 bp), Rx-1 (328 bp) and Xenk-2 (313 bp) (Fig. 8D; Supplementary Fig. S3).

## Discussion

### The intersect transcriptome identifies parasite contigs shared by fish and bryozoan hosts

Comparisons of host-specific expression of key genes may pave the road to targeted approaches to future disease control strategies and identify candidate genes for further functional characterisation. We developed an intersect transcriptome to identify a key set of *T.* *bryosalmonae* contigs that were expressed to at least some degree in both hosts, whilst minimising host contaminants. In line with our previous studies and existing myxozoan transcriptomes^18,23^, contigs in the intersect transcriptome were predominantly AT-rich (Fig. 1; 60–75% AT). The intersect transcriptome comprised a 7362 contig group, with a greater number of AT-rich contigs compared to the 5384 reciprocal contig group but possesses a greater proportion of non-AT rich contigs (Fig. 1). Although low, our Busco scores were comparable to previously reported CEG scores for myxosporeans^15^. It is well known that myxozoan genomes and transcripts are AT-rich (> 60% AT), and AT content can be used to identify transcripts of parasite versus host origin^15,18,23,35^. The increased representation of non-AT-rich contigs in the intersect may, therefore, reflect the presence of non-parasite sequences (see later discussion) but it may also reflect the presence of myxozoan transcripts that encode proteins rich in amino acids using GC rich codons, such as minicollagens^36^. Whilst parasite origin of certain non-AT rich cnidarian/myxozoan-specific transcripts may be identified, others would need to be PCR verified in the absence of a complete host-free parasite genome.

### Host-specific transcription indicates specialisation in nutrient acquisition and virulence

To identify host specific expression patterns, a subset of 52 contigs of specific interest to host–parasite interactions were chosen for RT-qPCR analysis in multiple infected fish and bryozoan samples. Primers designed to these contigs successfully amplified in both infected hosts and in multiple isolates and not in uninfected controls, confirming their parasite origin, along with their AT-rich composition. Proteases have been extensively targeted for therapeutic intervention due to their central roles in virulence mechanisms, including nutrient acquisition, cell/tissue invasion, and host immune evasion. Thus, they are essential to parasite survival and development in different host environments^25,37–40^. Parasite cysteine proteases are the most extensively studied group of proteases in parasites and, in this study, represent the second most abundant protease group in the intersect transcriptome (Supplementary Tables S1, S3). Host protein degrading CTS proteases are often co-expressed with nutrient acquiring and protease activating LGMN-like cysteine proteases in parasites^40^. Similar and high expression levels in both fish and bryozoan hosts of LGMN and CTSL imply that they are biologically important players in each respective host–parasite interaction (Fig. 5).

The dominance of metalloproteases in our *T.* *bryosalmonae* dataset is not surprising. Some of the most well characterised functional roles of parasite metalloproteases are prominent features of PKD pathogenesis, including modulation of the fish kidney tissue matrix and tissue remodelling that are most likely attributed to parasite migration, proliferation and differentiation^25,41^. Shedding of cell surface membrane proteins is fundamental to many cell signalling events triggered by changes in cellular microenvironments^25^. ADAM family members are an important group of ectodomain sheddases that regulate signalling associated with extracellular matrix homeostasis, often in association with tetraspanins. They regulate a wide range of functions including cell adhesion, inflammation, lymphocyte development, tissue invasion, and notch receptor processing. ADAM10 plays a central role in the regulation of immune and developmental events, including disease^25^. Consequently, dysregulation of ADAMs is strongly associated with infection-mediated tissue pathology and cancer, implicating them, along with tetraspanins, as important therapeutic targets^25,38,42–44^. In this study, we uncovered six ADAM-like transcripts, three that are ADAM10-like and three other contigs homologous to ADAM17, 22, and 28. Two of the ADAM10-like contigs exhibited a more bryozoan-specific expression profile suggesting that they may have an important regulatory role in bryozoan-parasite interactions (Supplementary Table S3, Fig. 5).

We have also uncovered several tetraspanins with a particularly abundant CD53-like transcript exhibiting a fish-specific expression profile (Supplementary Table S1, Fig. 8B). In mammals, tetraspanins in the CD family are important immune regulators with hematopoietic-specific CD53 being a suppressor of inflammatory cytokines^45,46^. Thus, association between *T.* *bryosalmonae* ADAM and tetraspanin homologues may, in part, be responsible for *T.* *bryosalmonae-*mediated disease pathology and dampening of fish pro-inflammatory responses observed during PKD^11,47^. Similarly, the T lymphocyte hormone TMSB4, dampens inflammatory processes by influencing TLR activity^48^. The very high expression levels of a *T.* *bryosalmonae* TMSB4 homologue in each host exceeding *Tb*RPL18, highlight its general biological importance in both hosts.

Parasites often depend on salvaging of host lipids to fuel growth and survival in a range of hosts, including fish^49–51^. It is, therefore, not surprising to find a repertoire of *T.* *bryosalmonae* phospholipases with the majority belonging to the PLA1 family (Supplementary Table S4). PLA1s hydrolyze acylglycerols and phospholipids, liberating free fatty acids and lysophospholipids^52^. All full-length PLA1 transcripts revealed in this study are likely to be secretory with most having highly fish-specific expression profiles. Importantly, PLA1A and Lipase H release the lipid mediators lysophosphatidylserine (LPS) and lysophosphatidic acid (LPA) that induce cell proliferation and chronic disease pathology^52,53^. It is, therefore, tantalizing to speculate that such lipid mediators may drive PKD pathology. PLA2 activity, can also lead to the release of these lipid mediators, albeit to a lesser extent than PLA1. We did not uncover any calcium dependent PLA2 family members that are linked to arachidonic acid release and production of inflammatory eicosanoids. All PLA2s we found were homologous to intracellular calcium independent PLA2s implicated, to a lesser extent, in immune regulation^54^. Interestingly, PLA2-G15 and PLAA were more highly expressed in bryozoans than in fish kidney tissue suggesting that these family members play a less important role in lipid metabolism of the parasite when in fish than in bryozoan hosts.

As in previous myxozoan studies, we uncovered several LDLR-A transcripts^18^ with 3 being highly fish-specific (Supplementary Table S4, Fig. 6). LDLR-A proteins are crucial in receptor-mediated endocytosis of ligands, particularly cholesterol and acyglycerols. Many parasites are unable to produce cholesterol and are, therefore, dependent on host acquisition to fuel cell proliferation and differentiation^51,55^. Consequent alterations in host cholesterol homeostasis can lead to immune dysregulation and disease pathology. The apparent dominance of myxozoan LDLR-As and PLA1 family members in infected fish kidney tissue is strongly indicative of host cholesterol and acyglycerol exploitation that could be a major contributory factor to PKD pathogenesis. Indeed, recent studies describe several viable pharmacological approaches to interfere with parasite utilisation of host lipids, which may have relevance to the future control of myxozoan parasites^56–58^.

### Host-specific transcription linked to parasite developmental processes

To develop a fuller understanding of myxozoan biology and potential future directions in disease mitigation, we examined datasets for genes involved in parasite development and functional morphology in each host. ADAM family members, especially ADAM10, are crucial in the regulation of notch signalling, implicating the involvement of some members in regulation of embryonic and adult developmental processes and thus as potentially important therapeutic targets^42,59^. The presence of meiosis in *T.* *bryosalmonae* stages in bryozoan hosts, may explain the bryozoan dominance of two of the ADAM 10-like molecules in this study.

Activation of natriuretic peptide receptors, including GC/NPR-A, leads to the accumulation of intracellular cGMP. They act as environmental sensors involved in the regulation of developmental events, blood pressure regulation, immune and electrolyte homeostasis in higher vertebrates^60^. The existence of NPR-like ligands in invertebrates is considered to be due to convergent evolution^61^. Whilst there are some functional similarities in the natriuretic systems of higher and lower vertebrates, such as ionic balance and osmoregulation, there is little known about this system in invertebrates^61^. Importantly, cyclic nucleotide balance, in general, is known to be important in parasite development, host-to-host transmission, target tissue recognition, and immune evasion events, enabling rapid adaptation to different host environments^62,63^. The significance of the bryozoan-specific nature of GC/NPR-A in this study may relate to the different parasite developmental events in each host or differences in electrolyte homeostasis and environmental sensing.

Heat Shock Proteins (hsps) have also been linked to both virulence and parasite development. They are crucial in maintaining protein homeostasis under extreme changes in environmental conditions and are, thus, essential to parasite survival, temperature-driven development and cell or tissue invasion^64,65^. Hsp60, 70, and 90 *T.* *bryosalmonae* homologues were found to be abundantly expressed in both hosts with Hsp60 and 90 exhibiting a bryozoan-specific expression profile. Since parasite developmental events in both hosts are known to be temperature dependent, bryozoan specificity of Hsp60 and 90 could be attributed to events unique to spore sac development, such as meiosis during sporogony^21,66^. Transcripts involved in cell–cell communication such as; neural sensory processing (Nrxn 4-like) and extracellular matrix interactions (MFAP4) exhibited host-specific expression profiles (Fig. 7). The expression of genes linked to neural development is intriguing given the lack of defined nervous systems in myxozoans^20^ and the inert nature of *T.* *bryosalmonae* sacs.

Minicollagens are likely to play a similar role to those in free-living cnidarians providing the high tensile strength needed to facilitate polar filament deployment^67^. In myxozoans this enables spores to attach to hosts. The dominance of minicollagen expression in bryozoan hosts that support spore development relative to the dead-end fish host is, therefore, an expected result now confirmed here by our expression analyses.

For the first time in myxozoans, we have detected several homeobox proteins and a frizzled receptor homologue (FZD-2). As in previous myxozoan studies, we did not find any Hox genes nor other elements of Wnt pathways. *T.* *bryosalmonae* FZD-2 and most homeobox proteins displayed bryozoan-specific expression profiles. This may further illustrate a developmental deficit in rainbow trout hosts (in contrast to natural fish hosts, such as brown trout, where viable spore development occurs). It is also possible that multicellular sac development in bryozoans requires more sophisticated regulation than cellular proliferation and development of simple pseudoplasmodia in fish hosts. Nevertheless, fish-specific expression of a Ceh11-like transcript suggests that some *T.* *bryosalmonae* homeobox proteins may have distinct roles in driving fish-specific development. We note, however, that caution must be exercised in assignment of *T.* *bryosalmonae* homeobox proteins to specific families. This requires in-depth phylogenetic analysis and will be facilitated when more family members are discovered in other myxozoans. Likewise, whether our frizzled receptor is truly indicative of Wnt pathways in myxozoans awaits clarification, particularly as they are also involved in non-Wnt signalling pathways^68^.

### Limitations and caveats

Limitations of this study include lack of insight on proteins expressed during normal development of *T.* *bryosalmonae* pseudoplasmodia in kidney tubules of natural fish hosts and in the cryptic stages that develop in association with the body walls of bryozoan hosts. A range of other proteins may be expressed during these phases of the life cycle of *T.* *bryosalmonae*.

Non-parasite (< 60% AT) sequences were evident in the blob plots of both the fish kidney-derived and spore sac-derived transcriptomes. As highlighted by Foox and colleagues^23^, this illustrates the inadequacy of a single step depletion of host sequences by BLAST analysis against fish sequence resources. Notably the second non AT-rich (< 60% AT) peak in the blob plot of the spore sac-derived transcriptome, relative to trout-derived and intersect transcriptome blob plots, was not substantially reduced following filtering using a set of partial *T.* *bryosalmonae* genomic contigs (Hartikainen pers comm). This is not surprising as both the genomic and transcriptomic contigs were derived from sequencing libraries prepared from parasite spore sacs isolated from bryozoans and likely carried some bryozoan tissue. Importantly, all spore sacs used in this study appeared to be host-free with no visible signs of host tissue. Nevertheless, despite this scrutiny, it is likely that host coelomocytes adhere to and spread over the surface of spore sacs and could account for some of the host sequence contamination. Indeed, it is likely that non-parasite sequences are universally present in existing myxosporean assemblies due to the presence of host cell/tissue contaminants^23^. We propose that in the absence of clean and complete host and parasite genomes to filter against, the intersect transcriptome approach can identify a key set of contigs for targeted analysis as shown in this study. Overall, the blob profiles provide a level of quality control for host contamination, given the known AT-rich (> 60% AT) nature of *T.* *bryosalmonae* contigs, relative to *ca*. 49% AT content of the recently published *F.* *sultana* transcriptome^69^.

The intersect transcriptome is, however, not assumed to represent a complete transcriptome. A perhaps more important limitation is that numerous parasite transcripts are likely to be amongst the apparent bryozoan-specific and fish-specific mismatches. Both sets of mismatched contigs are unlikely to reflect host exclusivity but rather low transcriptome coverage, limitations of de novo assembly, and limited availability of *T.* *bryosalmonae* resources. Yet, this can also be construed an advantage when comparative follow up studies are conducted, as tracking transcripts expressed in both hosts could help to unravel host-specific aspects of the parasite’s biology. Indeed, BLASTX annotation of both groups of mismatches enabled us to pinpoint potentially important *T.* *bryosalmonae* players in host–parasite interactions (eg. TMSB4, MMP, LGMN, ADAM10-3; Supplementary Table S5). In addition, all *T.* *bryosalmonae* homeobox proteins reported in this study were identified in the two groups of host-specific mismatches. Likewise, FZ-2 was identified in the bryozoan-specific group of mismatches (Table 2, Supplementary Table S5). Given the contentious nature of the existence of homeobox and Wnt signalling components in myxozoans, we additionally further confirmed parasite origin via the detection of each gene specifically in genomic DNA from infected hosts (Fig. 8D; Supplementary Fig. S3).

**Table 2:** Predicted developmental genes in *T.* *bryosalmonae* with closest manually annotated sequence by species given alongside predicted protein domains, namely; signal peptide (SP), frizzled (Fz) and smoothened (Sm).

Accession number	Gene name (contig number)	Closest species (accession no.)	E value (BlastX)	%AT	ORF (AA)	Protein domains/motifs
MN056840	Homeobox protein, Xenk-2 (C-2190)	*Amphibalanus* *amphitrite* (KAF0307692.1)	4e−17	75	196 (FL)	1 × Homeobox
MN056845	Homeobox protein, Anf-1-like (C-8582)	*Gallus gallus* (P79775)	5e−12	73	199 (FL)	1 × Homeobox
MN056841	Retinal homeobox-1 (Rx-1) (C-972)	*Mus musculus* (P80206)	9e−08	72	182 (FL)	1 × Homeobox
MN056842	Homeobox protein, OTX-2 (C-18561)	*Mus musculus* (P80206)	1e−13	68	345 (FL)	1 × Homeobox
MN056843	Homeobox protein, Sebox (C-4608)	*Callorhinchus milii* (XP_007894674)	6e−02	73	213 (FL)	1 × Homeobox
MN056844	Homeobox protein, Ceh11 (C-44466)*	*Caenorhabditis elegans* (P17486)	1e−12	71	93 (P)	1 × Homeobox
MN056859	Frizzled receptor-2 (C-2600)	*Caenorhabditis* *elegans* (G5ECQ2)	2e−17	72	613 (P)	Sp, 1 × Fz domain, 1 × Fz/Sm membrane region

Some 26% of contigs could not be annotated and thus encode unknown or orphan proteins. Comparable proportions of unknowns have been reported in other parasite transcriptomic datasets, including myxosporeans^18,23,70^. Myxozoans have undergone both extreme genome reduction and rapid molecular evolution and the high proportions of unknown proteins may reflect long branches that preclude resolving relationships to currently characterised proteins. We shortlisted seven unknown proteins for gene expression analysis that are representative of putative secretory and non-secretory unknowns found in the intersect transcriptome (C-168, C-660) and in the bryozoan-specific (C-1242) or fish-specific (C-28487, C-39373, C-51833, C-52333) groups of mismatches. Of the seven unknowns examined in this study, five exhibited host-specific expression profiles, implying that some unknown proteins may have particularly important host-specific functional roles in each host–parasite interaction. Given the extremely small size of myxozoan genomes compared to other metazoans, it is intriguing that they have retained such a large number of unknowns in their genomes/transcriptomes. Thus, it will be a major opportunity and challenge to determine how these unknown proteins may reflect intra- and inter-host adaptations to parasitism in myxozoans.

## Concluding remarks

We have generated the first insights on malacosporean gene expression based on the analyses of transcriptome datasets using a 2-host sequencing approach. Subsequent RT-qPCR analysis enabled us to gain novel insights on myxozoan biology, particularly regarding virulence, metabolism and development. This study will promote future work to understand and mitigate myxozoan-borne diseases.

## Materials and methods

### Bryozoan and rainbow trout kidney tissue sampling

Infected bryozoans (*F.* *sultana*) were collected in late spring from the River Cerne, Dorset, UK, as described previously^3^. Immature and mature parasites (spore sacs) were isolated by dissection from hosts, rinsed in fresh river water and checked to ensure there were no other tissues associated with each spore sac before placing directly into ice-cold Trizol-reagent (Sigma) by capillary action. 150 spore sacs were pooled and used to generate sufficient total RNA for cDNA library construction. Lysed spore sacs in Trizol-reagent were stored at − 80 °C prior to RNA extraction.

For gene expression analysis, uninfected and overtly infected bryozoans from the Furtbach River, Switzerland, were used. Bryozoan collections were cultured in a laboratory mesocosm, as described previously^71^, with successfully attaching bryozoan branches constituting a single colony. All bryozoan cultures were maintained for 1–3 weeks to allow colonies to develop transparency, enabling the detection of overt infections using a stereomicroscope. Uninfected bryozoan colonies were assessed based on colony morphology, absence of overt *T.* *bryosalmonae* spore sacs and by 18S rDNA-based qPCR analysis, as described previously^72^. Similarly, confirmation that each spore sac-containing bryozoan sample was, indeed, infected with *T.* *bryosalmonae*, was obtained by 18S rDNA and RPL18 qPCR and sequence verification, as described previously^33,72^. We were unable to distinguish between different stages of overt spore sac infection in bryozoans with only bryozoan colonies exhibiting clear spore sac infection used for gene expression analysis. To ensure sufficient total RNA for RT-qPCR analysis, 4–8 individual uninfected or overtly infected colonies were pooled to form a single biological replicate. In total, 3–4 biological replicates were created for each infection category, with each replicate placed into 1 ml RNAlater for 24 h at 4 °C and stored at − 80 °C prior to RNA extraction.

Uninfected and infected rainbow trout exhibiting advanced clinical disease (kidney swelling grade 2–3), as determined using the Clifton-Hadley grading system^73^, were sampled from a commercial trout farm located in Southern England with a history of annual PKD outbreaks, as described previously^33^. From parallel studies in our laboratory, examining the dynamics of *T.* *bryosalmonae* transcript expression at different stages of clinical disease, expression levels in fish kidney samples reached maximal thresholds at grades 2 and 3. Thus, only fish kidneys graded from 2 to 3 were considered in subsequent cDNA library preparation and gene expression analysis (M. Faber, unpublished data). Presence/absence of *T.* *bryosalmonae* was confirmed by 18S rDNA/ RPL18 qPCR and sequence analysis, with extrasporogonic parasite stages in kidney interstitial tissue confirmed by histological examination, as described previously^33,72^. Checks for other common bacterial pathogens, including *Aeromonas* *salmonicida*, were conducted using kidney swabs, as described previously^33^. Kidney swabs were streaked onto TSA plates (Becton–Dickinson, Oxford, UK). Plates were incubated for 48–72 h at 20–22 °C before examination for bacterial growth. Approximately 100 mg trunk kidney tissue was removed immediately below the dorsal fin from each fish and archived in 1 ml RNAlater, as outlined above.

### RNA extraction and sequencing

Total RNA from *T.* *bryosalmonae* spore sacs and infected kidney tissue in Trizol-reagent was extracted from Trizol-reagent according to the manufacturer’s instructions. Owing to the very limited quantity and availability of *T.* *bryosalmonae* spore sacs for RNA extraction, archived spore sac Trizol homogenate was processed incrementally in six batches generating two aliquots of total RNA (Supplementary Fig. S1). To maximize retrieval of total RNA, GlycoBlue co-precipitant (15 mg/ml; Thermofisher) was included into the isopropanol step (1.5 μl per RNA sample). Owing to the presence of PCR inhibitors in bryozoan tissue, bryozoan total RNA was treated with a PowerClean Pro RNA Clean-up kit (Cambio) to remove free and nucleic acid-bound PCR inhibiting substances. PCR inhibition has been highlighted as a potential cause of low representation of myxosporean transcripts in qPCR studies, especially based on invertebrate tissue samples^74^. In our hands, we have found the PowerClean Pro RNA Clean-up kit to be highly effective in releaving PCR inhibition without the need to dilute cDNA samples (J.W. Holland, unpublished data). All total RNA samples were also treated with Dnase-I using a TURBO DNA-free kit (Invitrogen), quantified using NanoDrop ND-1000 UV–Vis spectrophotometer and quality checked using an Agilent Bioanalyser 2100 or using a Shimadzu MultiNA microchip system.

cDNA library construction, RNA sequencing, filtering of trout-derived reads using rainbow trout genome and transcriptome databases, and de novo assembly was performed by Vertis Biotechnologie AG (Friesing, Germany). Total RNA from bryozoan-derived *T.* *bryosalmonae* spore sacs was confirmed to be of high quality using an Agilent Bioanalyser 2100 (RIN > 8.0) prior to dry ice shipment to Vertis Biotechnologie AG (Friesing, Germany). Immediately prior to cDNA synthesis and library construction, total RNA quality was further confirmed using a Shimadzu MultiNA microchip electrophoresis system based on the ratio of 28S rRNA/18S rRNA (Supplementary Fig. S1). The two aliquots of spore sac total RNA were combined with a total yield of 1.3 µg. Poly (A) + RNA was isolated, reverse transcribed into cDNA and amplified using the Ovation RNA-Seq System V2 kit (NuGEN Technologies Inc.) according to the manufacturer’s instructions and fragmented by sonication. After end-repair and ligation of TruSeq adapters, cDNA was amplified by PCR and normalized by controlled denature-re-association to ensure maximum *T.* *bryosalmonae* representation in the transcriptome assembly. cDNA quality at each step was checked using a Shimadzu MultiNA microchip electrophoresis system (Supplementary Fig. S1). Single-stranded cDNA was purified by hydroxylapatite chromatography by PCR amplification. Normalized cDNA was gel fractionated and fragments ranging from 300 to 500 bp in length were sequenced using an Illumina HiSeq 2000 machine producing paired end 100 bp read lengths.

To ensure maximum *T.* *bryosalmonae* representation in the trout pre-parasite filtered transcriptome, total RNA from 65 kidney tissue samples was isolated and subjected to RT-qPCR, as described previously^75^. Primers unique to *T.* *bryosalmonae* and rainbow trout RPL18 (Supplementary Table S5) were used to determine the most heavily infected kidney samples for cDNA library construction, as described previously^33^. ΔCq values varied from 17.21 (lowest parasite burden) to 5.19 (highest parasite burden). Equal amounts of total RNA were combined from 3 kidney samples (combined total RNA yield = 21 μg) with the lowest ΔCq values (5.19, 5.22, 5.95) and quality assessed prior to and during cDNA library construction (Supplementary Fig. S2) and poly (A) + RNA sequencing performed, as described above.

### Transcriptome assembly

Raw sequencing reads were quality filtered using a phred quality score cut-off of 33 in ASCII format in accordance with Sanger FASTQ format and a minimum length cut-off of 20 bp using trimming and sequence quality control tools within the CLC Bio Genomics Workbench (version 6.5.1). In the absence of a high-quality *T.* *bryosalmonae* genome scaffold, sequence reads were assembled de novo. In the case of the fish kidney-derived reads, to remove sequences having high sequence identities to rainbow trout, the sequences were mapped locally using megablast^76^, to rainbow trout genome^29^ and transcriptome assemblies^30^. After removal of trout sequences (e-value cut-off = 5e^-5^), assembly of high-quality unmapped reads was performed using the CLC Bio Genomics Workbench 6.5.1 at an optimized k-mer size. Assembled sequences were clustered with TIGCL^77^ and CAP3^78^ with a minimal overlap of 100 bp and identity cut-off of 95% to form unigenes. Sequence reads from the *T.* *bryosalmonae* spore sac library, were not host filtered due to the absence of a *F.* *sultana* genome assembly and were directly assembled into contigs without removal of any contaminating bryozoan reads. All sequence reads have been deposited at the European Nucleotide Archive (ENA) under the accession number PRJEB19471. Fish kidney-derived contigs were further filtered by dc-megablast (e-value cut-off = 5e^−3^) using two available myxozoan genome assemblies. Firstly, the current genome assembly of the myxosporean, *T.* *kitauei* (GCA_000827895.1) and, secondly, a *T.* *bryosalmonae* genome (MiSeq) sequence dataset generated from spore sac genomic DNA with sequenced reads obtained from a single MiSeq run that yielded 6,334,237 PRINSEQ cleaned paired reads with read lengths ranging from 35 to 251 bp and a total GC content of 28% (H. Hartikainen, B. Okamura, unpublished data). Contigs mapping to both parasite genome sequence datasets constituted the final trout-derived parasite transcriptome dataset (referred to as the trout parasite-filtered transcriptome; Table 1). Owing to the low-level filtering of *T.* *bryosalmonae* contigs with the *T.* *kitauei* genome, only the *T.* *bryosalmonae* genome sequence dataset was used to filter the spore sac-derived contigs (resulting dataset referred to as the bryozoan parasite-filtered transcriptome; Table 1). Transcriptome metrics were calculated using Quast (version 3.1)^79^, the completeness of each transcriptome assembly was assessed using BUSCO (version 3)^80^ and raw reads were re-aligned to the assembly using Bowtie (version 1.1.1)^81^. Reciprocal blast (dc-megablast) analysis (e-value cutoff = 5e^-3^) was undertaken to determine contigs in common with both transcriptome datasets (intersect contigs). Reciprocal blast has been implemented in previous studies to discriminate between target (sequences or closely related sequences found in two sequence datasets) from non-target sequences^82,83^.

For assessment and visualisation of contamination, taxonomic assignment of each contig was carried out using BlobTools (version 0.9.19) at each stage of transcriptome production^84^.

An *F.* *sultana* transcriptome, from uninfected zoids, has become available since completion of transcriptome assemblies in the current study^68^. Retrospective use of this new sequence resource to filter *F. sultana*-derived raw reads from the bryozoan-derived spore sac HiSeq library data revealed a very low number of mapped reads (0.82% of total reads). This very likely accounts for only a fraction of the *F.* *sultana* contaminating sequence reads in the spore sac HiSeq library. The *F.* *sultana* transcriptome has, therefore, not been incorporated into the transcriptome assembly pipeline. Conversely, the *F.* *sultana* transcriptome revealed a significant level of host-derived reads (20.76%) in the *T.* *bryosalmonae* genome sequence data. This further reinforces the importance of the reciprocal blast analysis to identify contigs in common with both host-derived transcriptomes.

### Transcriptome annotation

Annotation was performed on all contigs derived from *T.* *bryosalmonae* spore sacs and on myxozoan-filtered fish kidney-derived contigs using the Trinotate pipeline (version 2.0.2)^85^. Protein prediction was performed using TransDecoder (version r20140704) using default parameters^86^. Contigs were compared to Swiss-Prot and TREMBL (uniref90) databases^87–89^; downloaded March 2015 from www.uniprot.org) using NCBI BLAST + (version 2.2.27) with *E*-values more than 10^–3^ considered non-significant. Both sets of contigs were also subjected to BLASTX searches against the non-redundant (nr) nucleotide database (downloaded from www.uniprot.org on the 17-03-2015). All contigs predicted to be protein coding were submitted to signal-P (version 4.1)^87^, TMHMM (version 2.0c)^88^ and to HMMER (version 3.0)^89^ against the PFAM database (version 27.0 downloaded Jan 2015)^90^. RNAmmer (version 1.2) was used to predict the presence of any contaminating ribosomal RNA sequences^91^. Gene Ontology assignment was performed using eggNOG, and Swiss-Prot matches mapped to Gene Ontology classes using the mapping file: ftp://ftp.ebi.ac.uk/pub/databases/GO/goa/UNIPROT/gene_association.goa_uniprot.gz. GO classifications of intersect contigs were visualized using WEGO software^32^. Contig sequences were compared to the MEROPS (release 12.1; https://www.ebi.ac.uk/merops/download_list.shtml)^92^ database using BLASTX (version 2.2.31)^93^ with an e value cut-off of 1e−05 and a percent identity cut-off of 50%, with the most significant hit determined by the lowest e value.

### Real-time quantitative PCR

We employed RT-qPCR to reveal parasite transcriptional differences between the two hosts.

Total RNA representing uninfected/overtly infected bryozoans and uninfected/infected rainbow trout kidney tissue with a swelling grade of 2 (n = 3–4) was reverse transcribed into cDNA, as described previously^33^. Negative controls (without reverse transcriptase; −RT) were included to account for any residual DNA contamination.

Owing to limited amounts of bryozoan tissue, two batches of bryozoan and trout cDNA were utilised in this study providing 3 or 4 biological replicates per group. For bryozoan and trout cDNA, *ca* 1.5 µg and 5 µg of total RNA was reverse transcribed in 20 and 40 µl reactions respectively using a Revert Aid first-strand cDNA synthesis kit (Fermentas) according to the manufacturer’s instructions. Primers were designed to transcripts encoding proteins involved in; protein and lipid metabolism, developmental processes, cell–cell communication, cnidarian-specific structures or unknown/unannotated proteins (Supplementary Table S5). Primers were designed towards the 3′ end of each open reading frame aided by the considerable difference in codon usage between *T.* *bryosalmonae* and host genes (*ca*. 60–75% AT and 45–55% AT respectively). To facilitate primer design for some contigs, further sequence was obtained by anchored PCR and all primers tested for specificity and efficiency using a previously developed normalised full-length spore sac cDNA library constructed by Vertis Biotechnologie AG (Friesing, Germany). The library was constructed from decapped poly A + RNA from spore sacs. Following 5′ RNA adapter ligation, first strand cDNA was performed, and cDNA amplified using 5′ and 3′ (oligo dT) adapter primers. Prior to directional cloning into eukaryotic expression vector, pcDNA3.1 + (Invitrogen), cDNA was normalised as described above.

Primer melting (Tm) temperature and annealing temperature (Ta) were assessed using OligoAnalyzer3.1 (https://www.idtdna.com/calc/analyzer) and listed for each primer pair (Supplementary Table S5). Many *T.* *bryosalmonae* contigs were found to have repeat elements or very AT-rich regions. Thus, it was not always possible to develop primer pairs generating amplicons in the range 150–300 bp with sufficient GC:AT content and melting profile to allow efficient and specific q-PCR. All amplicons in this study were, therefore, within the range 149–422 bp. All RT-qPCR data was analysed using LightCycler 480 Software 1.5.1 (Roche). Each sample reaction consisted of 4 μl of sample cDNA and 6 μl of SYBR Green (Thermofisher) master mix containing 0.5 μM of each forward and reverse primer (Supplementary Table S5), as described previously^33^. Cycling conditions used were; denaturation at 95 °C for 10 min followed by 40 cycles of 95 °C for 30 s, 54–69 °C for 20–30 s and 72 °C for 30 s with fluorescence acquisition at 84 °C for 5 s. Melting curve analysis was performed after each PCR cycle between 72 and 94 °C to ensure only a single product had been amplified. Purified PCR amplicons were diluted in TE buffer, quantified using a NanoDrop 8000 Spectrophotometer, adjusted to 1 nM, and serially diluted as described previously^33^.

Following sequence verification of each PCR standard, primer efficiency and concentration of each gene transcript was calculated by reference to its standard curve, with efficiencies ranging from 1.80 to 1.96 (90–98%). Similarly, primers were designed for three selected reference genes to enable transcriptional normalization, namely; *Tb*RPL18, *Tb*RPL12, and *Tb*ELFα. *Tb*RPL12 has been shown to be a suitable reference gene for a range of invertebrate phyla, including cnidarians^94,95^. Likewise, *Tb*RPL18 and *Tb*ELFα have been shown to be highly consistent as reference genes in invertebrates^94,96^. To test stability of each reference gene within the same host, between hosts, and across different cDNA sets, the coefficient of variation (CV) of the Cq was calculated from the ratio of standard deviation to the mean and expressed as a percentage.

Relative quantification of *T.* *bryosalmonae* transcripts in each infected host was calculated using data from serially diluted reference DNA and normalized against the *T.* *bryosalmonae* reference gene, RPL18 in each qPCR run and expressed as arbitrary units, as described previously^33^. Relative expression calculated in this way allows only comparison of host expression profiles for individual transcripts. Fold difference was used to facilitate interpretation of expression differences between hosts, with infected fish kidney expression levels normalized to infected bryozoan levels using the delta-delta Cq method^97^.

In support of the validity and reliability of this RT-qPCR assay, our previous laboratory studies indicate that the unknown fish-specific secretory antigen, P14G8 is expressed at the protein level in fish kidneys from early (grade 0–1) to advanced (grade 2–3) clinical disease and closely correlates with P14G8 transcription in kidney samples (Holland et al., in preparation). In contrast, the much lower P14G8 expression in infected bryozoan samples was concomitant with a lack of detectable protein. The unknown secretory antigen C-39373, examined in this study, exhibited a similar fish-specific transcriptional profile as PG148 (Fig. 8C). The reliability of our RT-qPCR data is further supported by the apparent dominance of parasite LDL-R transcripts in myxozoan-infected fish tissues, as reported previously (Fig. 6; Supplementary Table S4)^18,19^.

### DNA extraction and further verification of gene origin

Genomic DNA was extracted from Trizol-reagent homogenate using a DNA back extraction buffer (BEB) and dissolved in TE buffer, as described previously^98^. To further verify that the *T.* *bryosalmonae* transcripts corresponding to homeobox genes and a frizzled receptor homologue were parasite in origin, PCR analysis was conducted on genomic DNA from uninfected and *T.* *bryosalmonae*-infected bryozoan and rainbow trout kidney tissues using validated primers designed to specifically amplify genomic DNA, as listed in Supplementary Table S5. All PCR amplicons were verified by sequencing.

### Statistical analysis

All relative expression data sets of *T.* *bryosalmonae* were log2 transformed prior to statistical analysis to determine differences of expression. Using the Mann Whitney U test, *T.* *bryosalmonae* gene expression was considered significantly different between hosts when *P* < 0.05. All statistical analyses were performed using GraphPad Prism (version 5.04, GraphPad Software Inc., La Jolla, USA). No statistical test was performed on the fold change expression analysis calculated by the delta-delta Cq method, which was performed to improve visualisation of expression differences calculated by relative expression.

## Supplementary information

Supplementary Information.

## Data Availability

Additional data that support the findings of this study are available in; (1) Figshare submission of Supplementary Tables S1-S5 (10.6084/m9.figshare.12951041) and Supplementary Figs. S1–S3. (2) European Nucleotide Archive (ENA) sequence submission under the accession number PRJEB19471. Raw sequence reads are available for both trout-derived and spore sac-derived transcriptomes. (3) Figshare submission of intersect transcriptome and bryozoan/trout pre- and post-parasite filtered *T.* *bryosalmonae* transcriptome datasets (10.6084/m9.figshare.11889672), (4) GenBank submission of individual contig sequences (accession numbers provided in Supplementary Table S5).
