# Supplementary material for: Comparative transcriptomics and host-specific parasite gene expression profiles inform on drivers of proliferative kidney disease
Source: Sci Rep. 2021 Jan 25;11:2149. doi: 10.1038/s41598-020-77881-7 (PMC7835236; doi:10.1038/s41598-020-77881-7)
Supplement: Supplementary file 1 — Supplementary Information. [file 41598_2020_77881_MOESM1_ESM.pdf]

Comparative transcriptomics and host-specific parasite gene expression profiles inform on drivers of proliferative kidney disease

Marc Faber[1], Sophie Shaw[2], Sohye Yoon[1,3], Eduardo de Paiva Alves[2,4], Bei Wang[1,5], Zhitao Qi[1,6], Beth Okamura[7], Hanna Hartikainen[8], Christopher J. Secombes[1], Jason W. Holland[1 *]

[1] Scottish Fish Immunology Research Centre, University of Aberdeen, Aberdeen AB24 2TZ, UK.

[2] Centre for Genome Enabled Biology and Medicine, University of Aberdeen, Aberdeen AB24 2TZ, UK.

[3] Present address: Genome Innovation Hub, Institute for Molecular Bioscience, The University of Queensland, Brisbane, QLD 4072, Australia.

[4] Aigenpulse.com, 115J Olympic Avenue, Milton Park, Abingdon, Oxfordshire, OX14 4SA, UK.

[5] Guangdong Provincial Key Laboratory of Pathogenic Biology and Epidemiology for Aquatic Economic Animal, Key Laboratory of Control for Disease of Aquatic Animals of Guangdong Higher Education Institutes, College of Fishery, Guangdong Ocean University, Zhanjiang, P.R. China.

[6] Key Laboratory of Biochemistry and Biotechnology of Marine Wetland of Jiangsu Province, Yancheng Institute of Technology, Jiangsu, Yancheng, 224051, China.

[7] Department of Life Sciences, The Natural History Museum, London SW7 5BD, UK.

[8] School of Life Sciences, University of Nottingham, Nottingham, NG7 2RD, UK.

[*] Author for correspondence.

Tel: +44 1224 438047

Email: j.holland@abdn.ac.uk

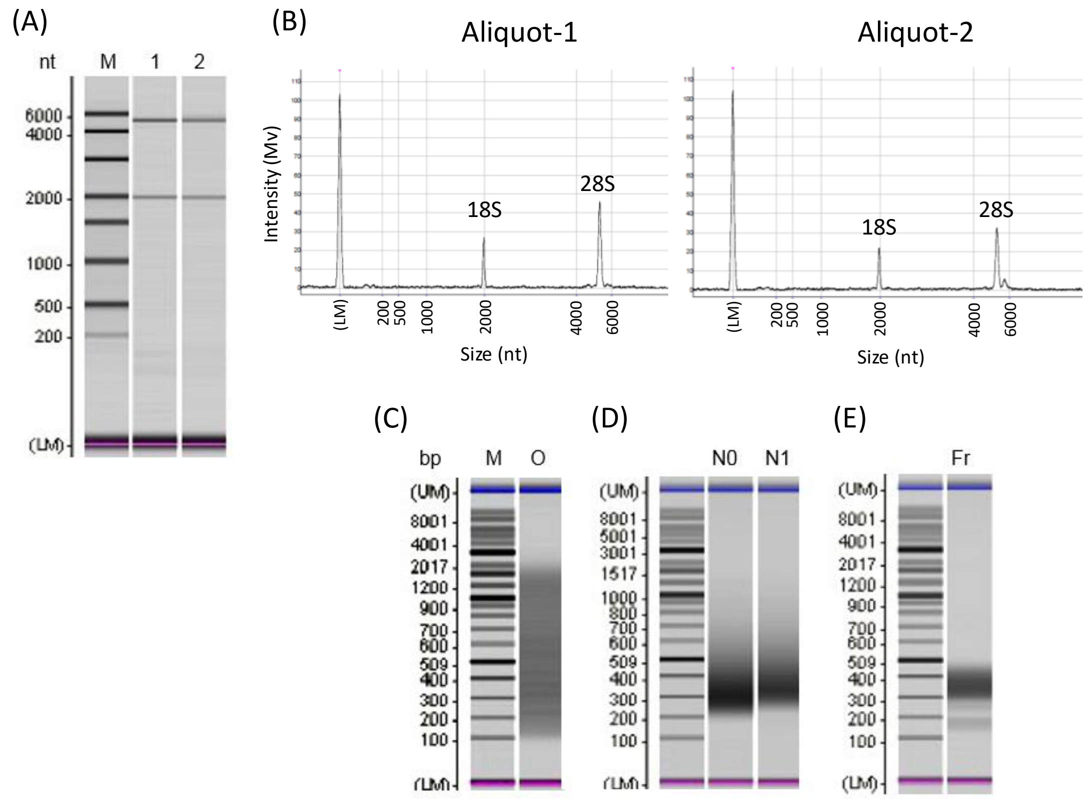

Supplementary Figure S1. Shimadzu MultiNA microchip total RNA and cDNA electrophoresis profiles prior to and during Illumina cDNA library preparation. (A) Spore sac total RNA (total yield = 1.3 µg supplied as 2 aliquots (Aliquot-1 & Aliquot-2) of 0.6 & 0.7 µg in 64 µl and 71 µl RNase-free water respectively) were used to generate the bryozoan-derived spore sac Illumina library. (B) Electropherograms representing the two total RNA aliquots used for cDNA library preparation with 28S rRNA / 18S rRNA ratios of 1.6 (RNA-1) and 1.4 (RNA-2). (C) Poly A+ RNA was purified, and reverse transcribed into cDNA using the Ovation RNA-Seq System V2 kit (NuGEN Technologies Inc). (D-E) Amplified cDNA was fragmented with ultrasound (2 pulses of 30 s at 4°C), end repaired, and TruSeq sequencing adapters ligated to the cDNA fragments. cDNA normalisation was undertaken by one cycle of denaturation and reassociation of the cDNA. Single stranded cDNA was separated from double stranded cDNA by hydroxylapatite chromatography. Normalised cDNA was size fractionated (size range 300-500 bp) using a differential clean-up with an AgenCourt AMPure kit prior to end repair and adapter ligation. M = Size marker. O = cDNA generated using the Ovation RNA-Seq System V2 kit. NO = PCR-amplified cDNA. N1 = normalised cDNA. Fr = size fractionated N1 cDNA.

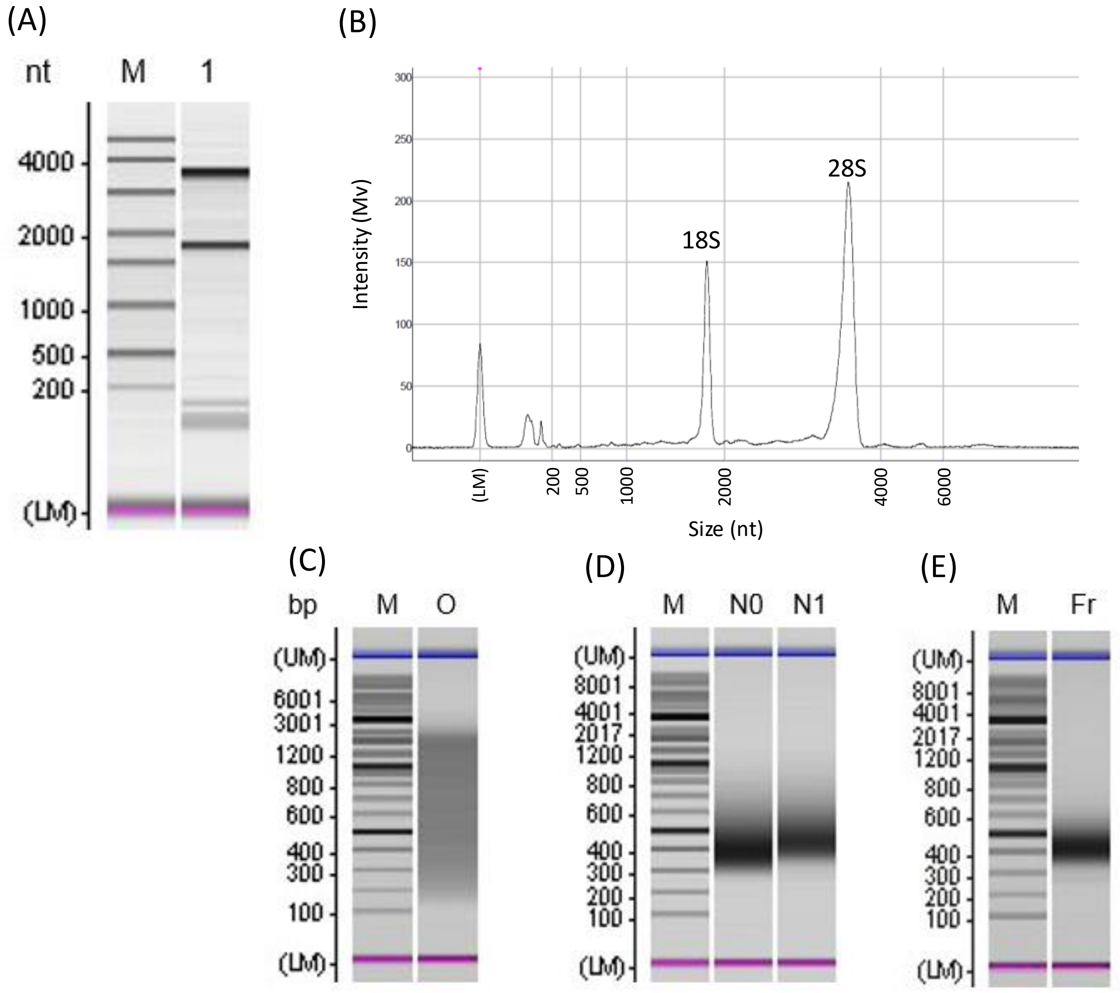

Supplementary Figure S2. Shimadzu MultiNA microchip total RNA and cDNA electrophoresis profiles prior to and during Illumina cDNA library preparation. (A) Fish kidney total RNA (total yield = 21 μg in 42 μl RNase-free water) used to generate the fish-derived Illumina library. (B) Electropherogram representing fish kidney total RNA used for cDNA library preparation with a 28S rRNA / 18S rRNA ratio of 1.9. (C) Poly A+ RNA was purified, and reverse transcribed into cDNA using the Ovation RNA-Seq System V2 kit (NuGEN Technologies Inc). (D-E) Amplified cDNA was fragmented with ultrasound (2 pulses of 30 s at 4°C), end repaired, and TruSeq sequencing adapters ligated to the cDNA fragments. cDNA normalisation was undertaken by one cycle of denaturation and reassociation of the cDNA. Single stranded cDNA was separated from double stranded cDNA by hydroxylapatite chromatography. Normalised cDNA was size fractionated (size range 300-600 bp) using a differential clean-up with an AgenCourt AMPure kit prior to end repair and adapter ligation. M = Size marker. O = cDNA generated using the Ovation RNA-Seq System V2 kit. NO = PCR-amplified cDNA. N1 = normalised cDNA. Fr = size fractionated N1 cDNA.

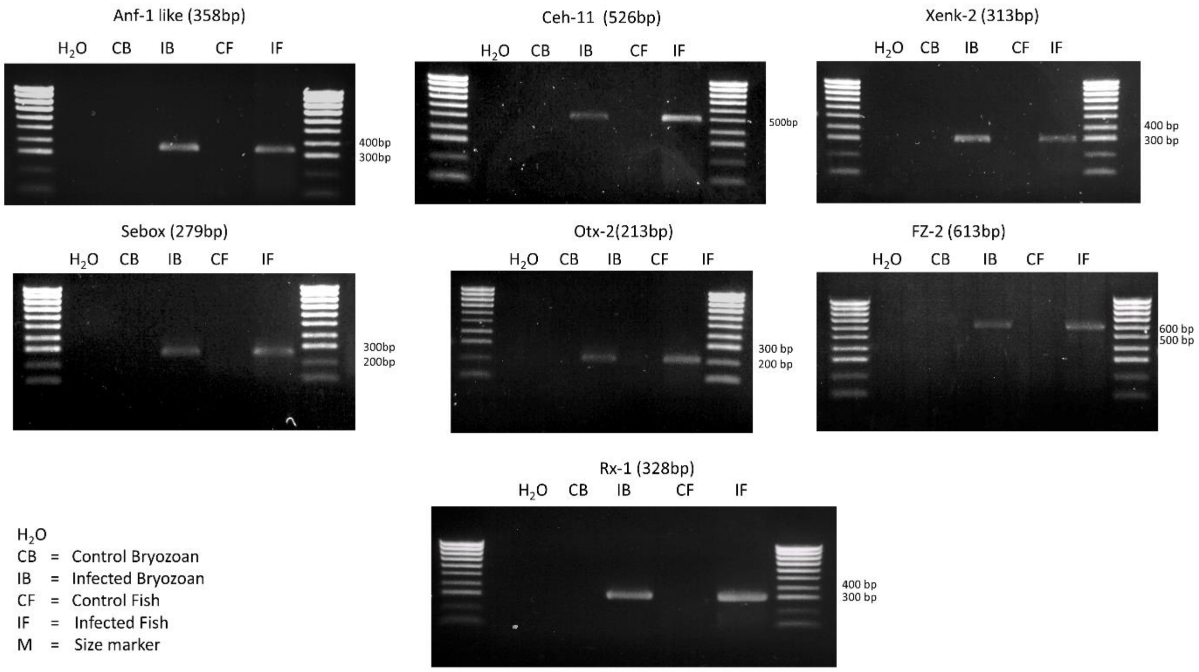

Supplementary Figure S3. Uncropped pictures of the PCR profile shown in Figure 8D. Validation of Sebox, Ceh-11, Anf-1-like, Otx-2, FZ-2, Rx-1 and Xenk-2 in negative control (H_20), uninfected bryozoan and fish kidney controls respectively (CB & CF) and *T. bryosalmonae* infected bryozoan and fish genomic DNA respectively (IB & IF). All blots are flanked by the Molecular weight marker.
